# Tuning the Photophysical Properties of BODIPY Dyes and Studying Their Self-Assembly via Hydrogen Bonding

**DOI:** 10.1021/acsomega.4c09745

**Published:** 2024-12-27

**Authors:** Büşra Akyol, Eylül Merve Çokluk, Mehmet Menaf Ayhan, Sinem Tuncel Kostakoğlu, Ayşe Gül Gürek

**Affiliations:** Department of Chemistry Gebze Technical University, Gebze Kocaeli 41400, Turkey

## Abstract

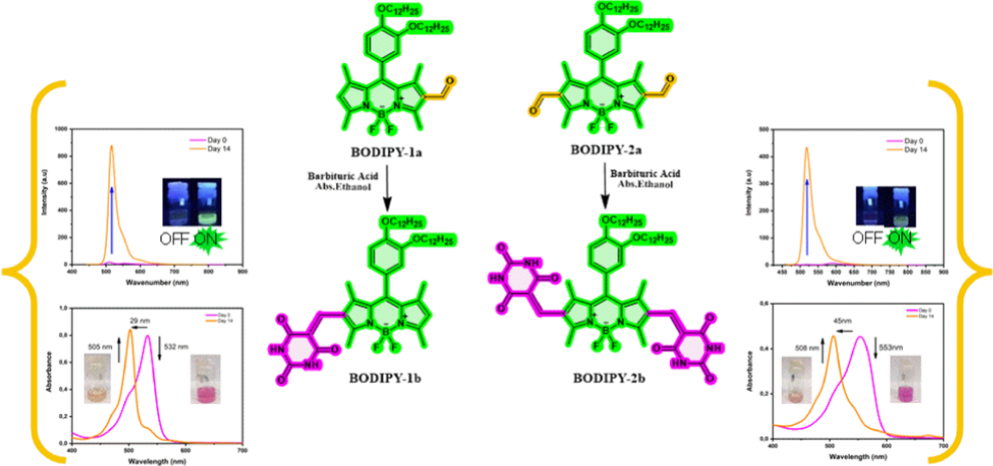

Here, BODIPY derivatives were functionalized with barbituric acid, which has multiple hydrogen bonding abilities that are directional, to have highly ordered hydrogen bond–mediated self-assembled structures to tune BODIPY’s photophysical properties. The synthesis of barbituric acid-functionalized BODIPY derivatives via Vilsmeier and Knoevenagel reactions was achieved, and the resulting compounds were characterized with FT-IR, ^1^H NMR, ^13^C NMR spectroscopy, and mass spectrometry. Hydrogen bond-mediated self-assembled structures were investigated through UV–vis and fluorescence spectrophotometry, ^1^H NMR spectroscopy, and a dynamic light scattering method in solution. Moreover, SEM, HR-TEM, and PXRD were used to study the self-assembly of compounds in bulk.

## Introduction

Supramolecular chemistry has emerged as a powerful tool to organize functional chromophores into supramolecular motifs via noncovalent interactions, including hydrogen bonding,^1^ halogen bonding,^2^ metal coordination,^3,4^ electrostatic interaction,^5^ π–π stacking, and the hydrophobic effect. These noncovalent interactions allow small molecules to self-assemble, resulting in molecular assemblies. Self-assembly involves spontaneously combining two or more molecules or ions to form larger aggregates with reversible supramolecular interactions. Over the past few decades, the concept of self-assembly has focused attention on creating various structures with diverse functions.

Hydrogen bonding is one of the most widely used noncovalent interactions and plays a significant role in designing supramolecular architectures, thanks to its strength and high directionality degree. A single hydrogen bond is too weak to drive the formation of supramolecular structures. Multiple hydrogen bonds are needed to increase the strength and directionality. Barbituric acid is a very suitable tool to build multiple hydrogen bonds.^6^ Besides, barbituric acid has an active methylene group and can easily undergo condensation reactions with conjugated aldehydes without a catalyst, followed by simple filtration to purify the resulting compound. This easily applicable synthetic procedure also makes barbituric acid a good candidate as a starting material. Yagai et al. have developed a variety of barbiturate π-conjugated molecules^7,8^ that can form six-membered supermacrocycles, called “rosettes,” through hydrogen bonds.^9,10^ Barbituric acid-functionalized oligothiophenes,^11−13^ naphthalenes,^14^ anthracenes,^15^ and oligo(p-phenylenevinylene)s^16^ were developed and self-assembled into nanoscopic structures via the formation of rosettes.

4,4-Difluoro-4-bora-3a,4a-diaza-s-indacene (BODIPY) dyes attract great attention due to their strong visible light absorption, high fluorescence emission,^17,18^ high stability to environmental conditions such as pH and solvent polarity, high fluorescence quantum efficiency, and low photobleaching since they were reported by Treibs and Kreuzer in 1968.^19^ Although BODIPYs were initially used in biological labeling,^20^ they have found place in many different application areas such as photodynamic therapy,^21^ bioimaging and sensing,^22−24^ drug delivery systems,^25^ medical treatment,^26^ molecular logic gates,^27^ photothermal therapy,^28^ photovoltaics,^29^ light-harvesting systems,^30^ and liquid crystals^31^ by adjusting their optoelectronic properties, thanks to the significant advances achieved in the functionalization of the BODIPY skeleton. The absorption and emission characteristics of the BODIPY can be adjusted by structural modification and self-assembly pathways.^32^ They can form aggregates through noncovalent interactions.^33^ Two types of aggregates are well-known: H- and J-aggregates. If monomers build a head-to-tail arrangement, the only transition to the lowest energy excited state (S_1_) is allowed and named as J-aggregates, showing intense, narrow, redshifted (bathochromic) absorbance. On the other hand, monomers build face-to-face structures in only an excited state, and the highest level of electronic transition is allowed, so H-aggregates demonstrate a hypsochromic (blue) shift in absorption spectra. Zhang et al. observed self-assembly nanowires of J-aggregated uracil-functionalized BODIPYs in polar solvents.^1^ Nevertheless, the supramolecular behavior of BODIPY dyes has been less investigated compared to other π-conjugated structures.^5,34^ In this study, two barbituric acid-functionalized BODIPY derivatives were prepared via the Knoevenagel condensation reaction. To the best of our knowledge, both barbituric acid-substituted BODIPY derivatives are novel, as well as their formyl derivatives, giving us the opportunity to study the self-assembly of BODIPY dyes. The aggregation behavior of compounds was evaluated in polar and nonpolar media. By providing control over the aggregation type, we can tune the optical properties for targeted applications, such as imaging, energy transfer, and sensing. The self-assembly of BODIPY was investigated in solution and the solid state.

## Results and Discussion

### Design, Synthesis, and Characterization

**Scheme 1:**
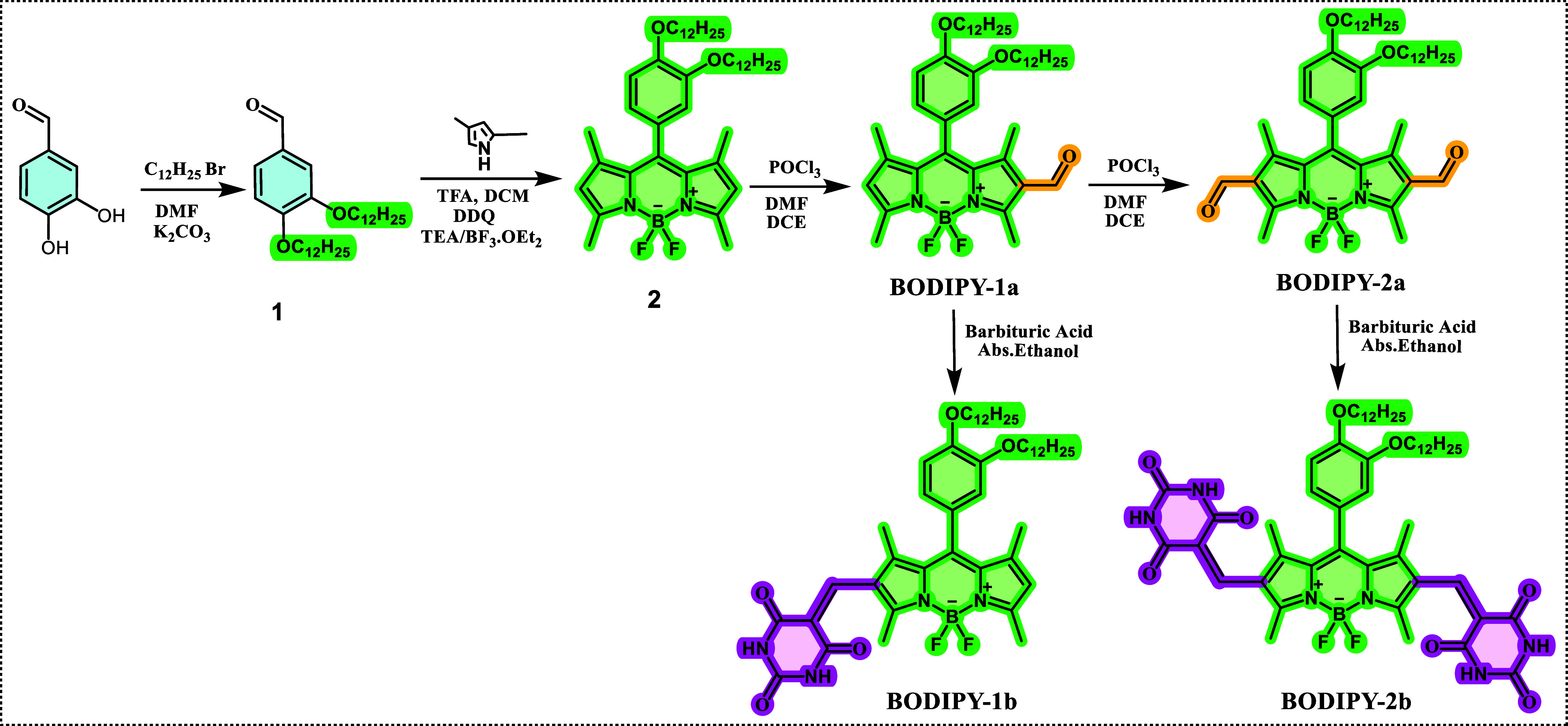
Synthesis of BODIPY Derivatives

Controlling molecular interactions during the self-assembly duration is a main source of problems that cause unexpected photophysical characteristics. To address this issue, sterically bulky substituents can be introduced to the chromophore’s core. Moreover, alkyl groups at the meso position of a BODIPY core provide good solubility in organic solvents for further modifications. Additionally, to achieve hydrogen bond-mediated self-assembled structures, long alkyl rings are needed. Initially, bulky 3,4-dihydroxybenzaldehyde was alkylated with 1-bromodecane to obtain compound **1** according to the literature,^35^ as shown in Scheme 1. Meso-substituted BODIPY core (compound **2**) was obtained by a condensation reaction between 2,4-dimethyl pyrrole and synthesized aromatic aldehyde derivative compound **1** in the presence of trifluoroacetic acid as an acid catalyst. In this step, purification was not applied because the obtained product was unstable. DDQ was used as an oxidant, and then, dipyrrin was obtained and reacted with boron trifluoride diethyl etherate in a basic medium provided by trimethylamine to pull off acidic hydrogen from nitrogen atoms. A fluorescent orange compound was obtained.

To have a high degree of internal order self-assembled structures, specific functional groups such as barbituric acid, which can engage in directional noncovalent forces like hydrogen bonding, were preferred to add to our molecular design. Barbituric acid’s intermolecular hydrogen bonding (N–H----O) ability demonstrates high aggregation capability in many works of literature. Subsequently, formyl groups were substituted to the BODIPY core, so barbituric acid could get involved in the Knoevenagel condensation reaction. The 2–6 positions are less positively charged, so they can be exposed to electrophilic attack. First, the tetramethyl BODIPY core was functionalized with the Vilsmeier reagent to obtain 2-formyl BODIPY (**BODIPY-1a**).^36^ Compound **BODIPY-2a,** having diformyl groups in the 2–6 positions of the BODIPY core, was obtained using the Vilsmeier reagent and compound **BODIPY-1a**. Finally, via the Knoevenagel condensation, which is a type of nucleophilic addition reaction, mono- (**BODIPY-1b**) and dibarbituric acid (**BODIPY-2b**)-substituted products were obtained.

The characterization of compounds was confirmed by molecular ion peaks in MALDI-TOF, FTIR, ^1^H, and ^13^C NMR spectroscopy (Figures S1–S18). Comparison of ^1^H NMR spectra of **BODIPY-1a** and **BODIPY-2a** shows the aldehydic proton intensity doublet in **BODIPY-2a**. Carbonyl peaks were observed at 1664 and 1673 ppm in FTIR spectra and carbonyl carbons at 185.97 and 185.69 ppm in the ^13^C NMR spectra for these compounds, respectively. The disappearance of aldehydic protons of compounds **BODIPY-1a** and **BODIPY-2a** resonating at 10.02 and 10.06 ppm with the simultaneous appearance of newly formed CH protons showing at 8.45 ppm for **BODIPY-1b** and 8.20 ppm for **BODIPY-2b** and also exchangeable NH protons proves the synthesis of **BODIPY-1b and BODIPY-2b**. To label exchangeable NH protons of molecules, deuterated water was added; they resonated at 8.35 and 8.03 ppm for **BODIPY-1b** and 10.43 and 10.29 ppm for **BODIPY-2b** (Figures S11 and S16). Additionally, the MALDI-TOF mass spectrum revealed molecular ion peaks at 830.75 [M]^+^, 812.58 [M+H–F]^+^ (**BODIPY-1b**) and 969.19 [M]^+^, 992.19 [M + Na]^+^, and 950.26 [M-F]^+^ (**BODIPY-2b**) (Figures S13 and S18).

### Photophysical Properties

According to UV–vis data in THF (Figure S19), all **BODIPY** derivatives show narrow spectra with two maxima, which are a shoulder and a sharp peak, corresponding to allowed S_0_-S_1_ (π–π*) transitions of the π-conjugated delocalized core and their vibrational outstanding structures at 500, 497, 504, 534, and 564 nm, as expected, since BODIPY dyes are characterized by strong absorption bands between 500 and 580 nm.^37^ Second absorption peaks between 330 and 470 nm are attributed as transitions from the ground state to a higher excited state S_0_-S_2_.^37^**BODIPY-1b** and **BODIPY-2b** are conjugated because of the substitution of barbituric acid. The extension of the conjugation system causes shifts in the spectrum toward longer wavelength (lower frequency, lower energy) absorptions as expected. Their molar absorptivities were calculated in THF as 133 100 (λ_max_ = 496 nm), 132 242 (λ_max_ = 503 nm), 91 600 (λ_max_ = 533 nm), and 115 757 (λ_max_ = 563 nm) L·mol^–1^·cm^–1^, respectively, because all products are highly soluble in THF (Figure S20).

Photophysical properties of **BODIPY-1b** and **BODIPY-2b** were examined in different solvents to investigate if there was any change in solutions depending on solvent polarities (Figure S21). Absorption of **BODIPY-1b** was hardly blueshifted when the solvent was changed from carbon tetrachloride and chloroform (λ = 542 nm) to acetonitrile (λ = 528 nm). **BODIPY-2b** dissolved in a few solvents, in contrast to **BODIPY-1b**, which were NMP, acetone, ethanol, ethyl acetate, THF, and 1,4-dioxane, and it showed blueshifted absorption peaks from 565 nm (1,4-dioxane) to 556 nm (NMP).

**Figure 1:**
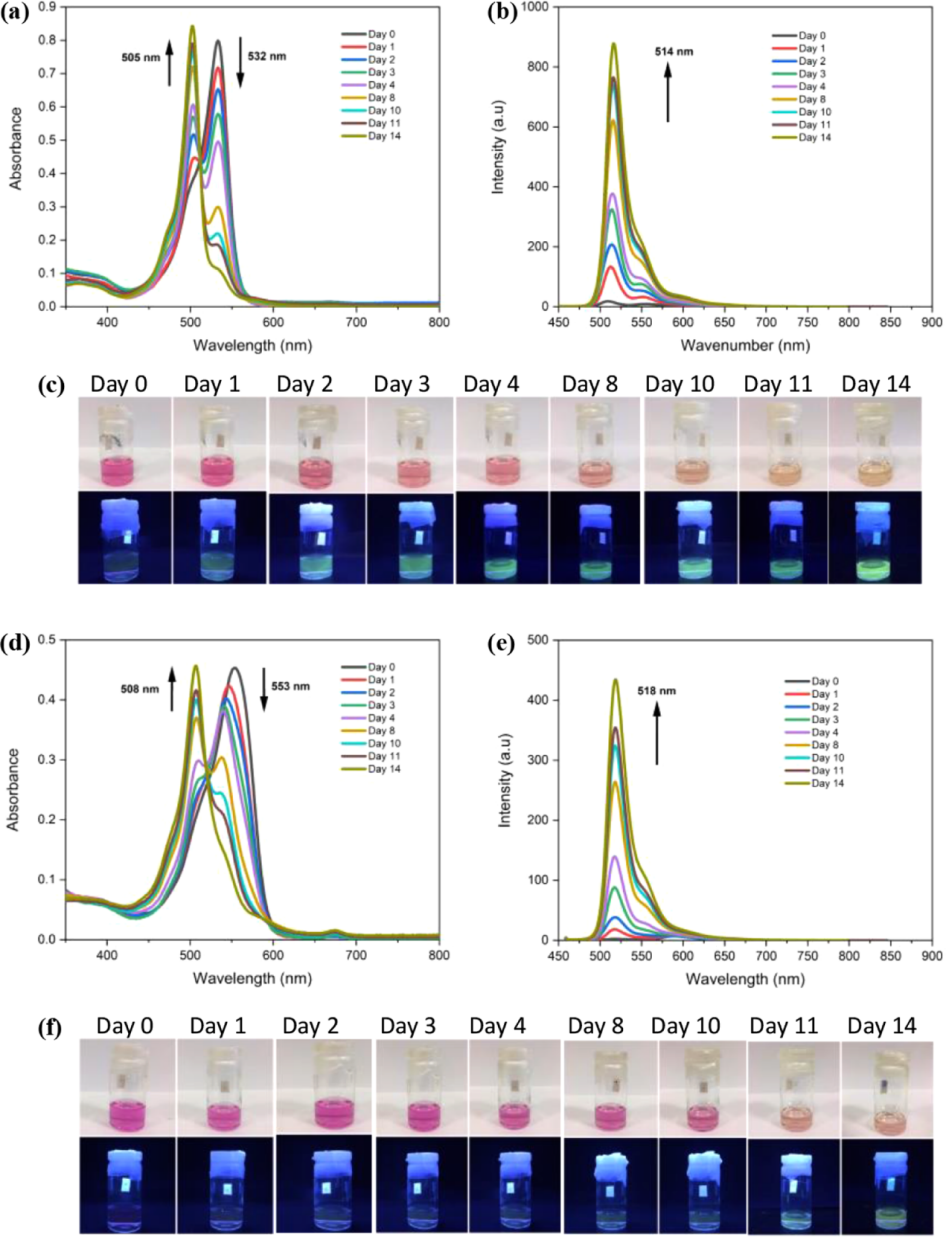
Time-dependent UV–vis absorption spectra of (a) **BODIPY-1b** and (d) **BODIPY-2b**; time-dependent fluorescence spectra of (b) **BODIPY-1b** (λ_ex_ = 435 nm) and (e) **BODIPY-2b** (λ_ex_ = 455 nm); time-dependent photographs of (c) **BODIPY-1b** and (f) **BODIPY-2b** in ethanol (10^–5^ M) taken under daylight and UV lamp (λ_ex_ = 365 nm) in ethanol solution (10^–5^ M).

It has been demonstrated that, according to the substituents of the BODIPY core, structures tend to constitute either parallel J-aggregates with redshifted absorption or blueshifted absorption antiparallel H-aggregates.^38^ To study the aggregation properties of barbituric acid-substituted BODIPY derivatives (**BODIPY-1b** and **BODIPY-2b**), time-dependent UV–vis absorption and fluorescence analysis were done in ethanol (Figure 1). The absorption band of **BODIPY-1b** was observed at 534 nm, which gradually shifted over time. After 15 days, the aggregation band at 534 nm was totally blueshifted by 29 nm to 505 nm (Figure 1a). Similarly, the absorption band of **BODIPY-2b** at 553 nm was blueshifted by 45 nm to 508 nm after 15 days (Figure 1d). A color change from pink to yellow was also observed during the gradual shift for both compounds (Figure 1c,f). The hypsochromic shift indicates an H-type of aggregation for **BODIPY-1b** and **BODIPY-2b**.

The fluorescence maximum of **BODIPY-1b** and **BODIPY-2b** was observed at 514 and 518 nm, respectively, in ethanol with a very low intensity, showing that both compounds are barely nonemissive in ethanol (Figure 1b,e). In general, the emission of the chromophores is quenched via H-type aggregates.^39^ However, a gradual increase in emission was observed for **BODIPY-1b** and **BODIPY-2b** in ethanol over time. After 15 days, the emission enhanced approximately 170 times for both compounds. It could be assumed that **BODIPY-1b** and **BODIPY-2b** form H-type aggregates in ethanol, causing emission enhancement that contrasts with quenched aggregation, which is typical for BODIPY dyes.^40^ This significant increase in emission can be due to aggregation-induced emission, a photophysical phenomenon in which a certain group of luminescent materials becomes highly luminous when aggregated in a poor solvent or solid state.^41^ It should also be noted that the compounds are nonemissive in the solid state.

**Figure 2:**
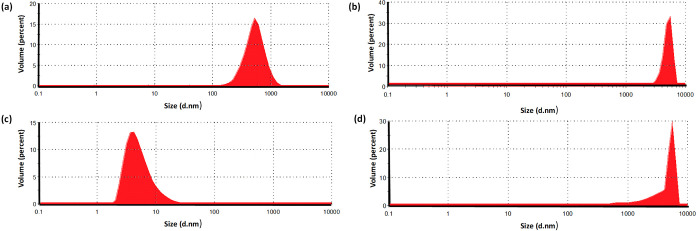
DLS size distribution (by volume) of BODIPY derivatives measured in ethanol solution (10^–5^ M) on the first day [(a) **BODIPY-1b**, (c) **BODIPY-2b**] and after 15 days [(b) **BODIPY-1b**, (d) **BODIPY-2b**].

To support aggregation formation, the DLS method was used to determine the size distribution of **BODIPY-1b** and **BODIPY-2b** (c = 10^–5^ M) at RT (Figure 2). Initially, compounds were measured in THF, in which the compounds are monomeric. After 15 days, data were repeated in ethanol. The data revealed detectable large aggregates (diameter greater than 10 nm) in ethanol for both compounds, confirming aggregation formation, while there were not any huge aggregates in THF.

**Figure 3:**
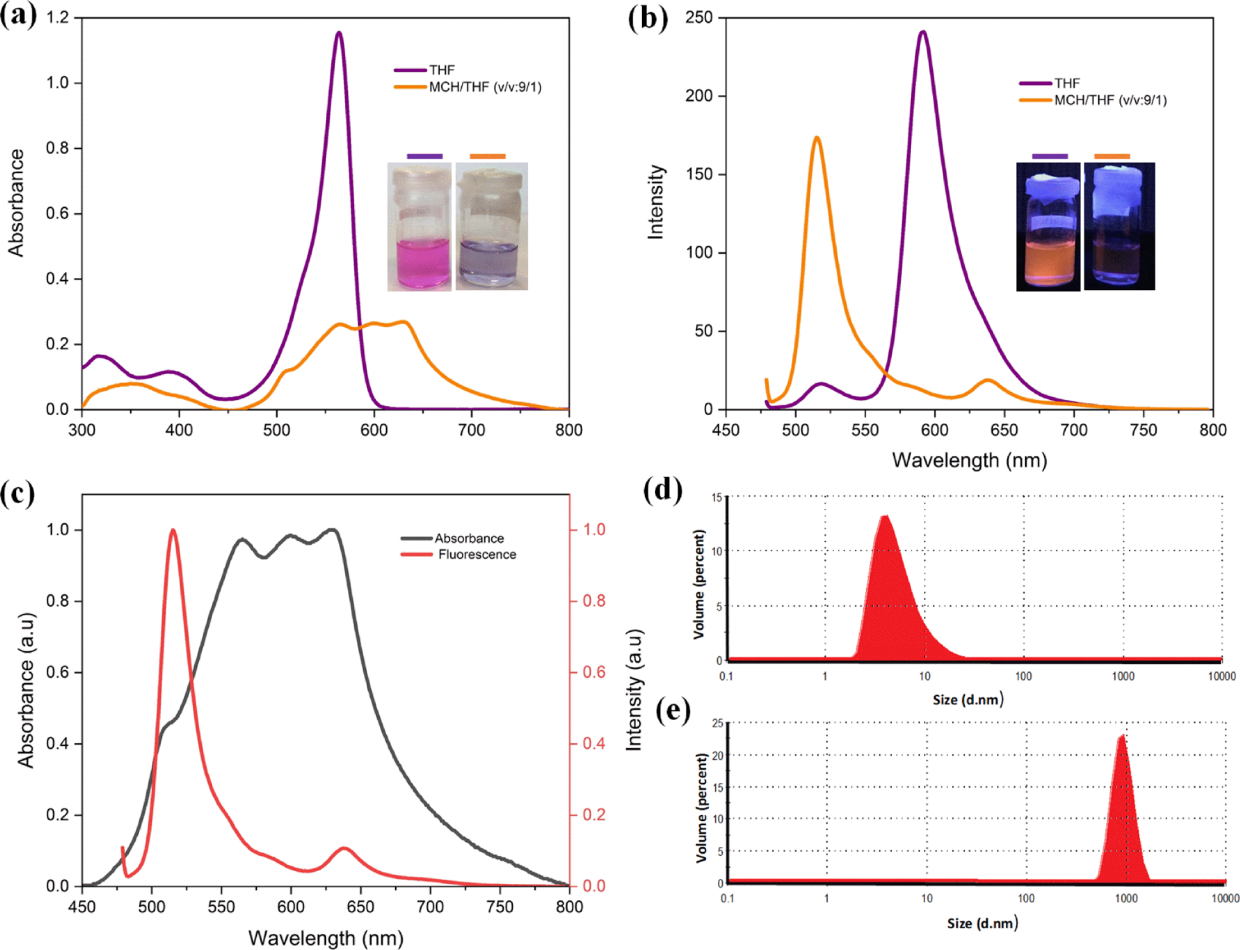
(a) UV–vis spectra of **BODIPY-2b** (10^–5^ M) in THF and MCH/THF (v/v:9/1), (b) their fluorescence spectra (λ_ex_ = 470 nm), (c) Stokes shift for MCH/THF (v/v:9/1), and DLS size distribution of **BODIPY-2b** in (d) THF and (e) MCH/THF (v/v:9/1).

According to studies of Yagai et al., different solvent systems were tried to observe the change in photophysical properties of compounds through their self-assembled structures.^42^**BODIPY-2b** is highly soluble in THF but scarcely soluble in nonpolar solvents such as methylcyclohexane (MCH). The effect of MCH on the self-assembly (c = 1 × 10^–5^ M) in THF was investigated by UV–vis, fluorescence, and DLS to demonstrate a solvent-induced effect of complexity in supramolecular systems based on various hydrogen bonds (Figure 3). Since **BODIPY-1b** is highly soluble even in nonpolar solvents like MCH, a study could not be done for this compound.

**BODIPY-2b** showed a maximum absorption at 564 nm in THF. When nonpolar solvents were added, there was redshifted absorption introducing parallel J-type aggregation (Figure 3a). The color changed from pink to purple. Moreover, the emission wavelength shifted from 591 to 514 nm (Figure 3b). There is fascinating fluorescence data observed in a high proportion of MCH as anti-Stokes photoluminescence (Figure 3c), which is also called up-conversion, in which photoemission energies will be higher than excitation energy.^43^ The DLS data of **BODIPY-2b** showed large aggregates in MCH/THF (v/v:9/1), confirming aggregation formation (Figure 3e).

### Concentration-Dependent ^1^H NMR

Concentration-dependent ^1^H NMR measurements have been applied in the literature to examine rosette or tape-like structures of barbituric-functionalized self-assembly via hydrogen bonding.^44^ To this aim, the hydrogen-bonded self-assembly of **BODIPY-1b** is interpreted with concentration-dependent ^1^H NMR in CDCl_3_. Unfortunately, the poor solubility of **BODIPY-2b** in CDCl_3_ was the obstacle to studying concentration-dependent ^1^H NMR spectroscopy for **BODIPY-2b**.

**Figure 4:**
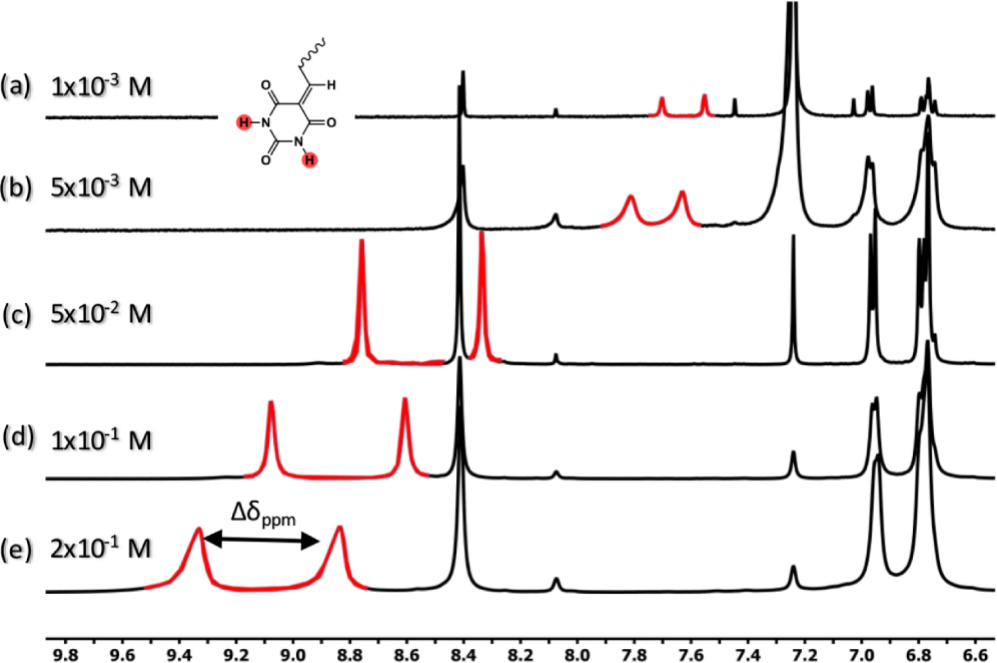
Concentration-dependent ^1^H NMR (500 MHz) of **BODIPY-1b** (c = 1 × 10^–3^ to 2 × 10^–1^ M) in CDCl_3_.

When the concentration is increased from 1 × 10^–3^ to 2 × 10^–1^ M, two NH signals in barbituric acid administrate a downfield shift, suggesting intermolecular hydrogen bonding (Figure 4a–e). Chemical shifts’ difference between two NH signals enhances with increasing concentration (Δδ_ppm_ = 0.15 ppm for c= 1 × 10^–3^ M, Δδ_ppm_ = 0.48 ppm for c = 2 × 10^–1^ M). This phenomenon is a characteristic of cyclic hydrogen-bonded rosette formation according to studies in the literature mentioned before.^44,45^

### Morphology

**Figure 5:**
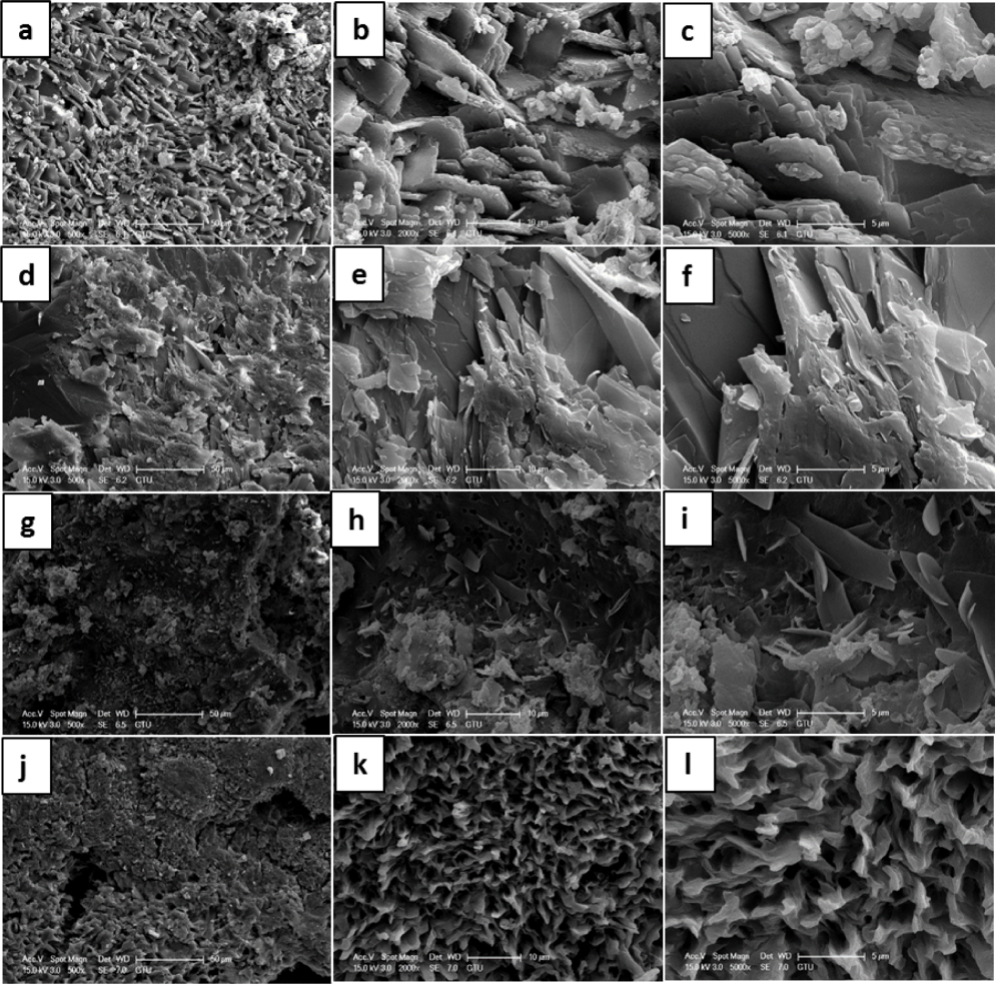
SEM images of (a–c) **BODIPY-1a**, (d–f) **BODIPY-2a**, (g–i) **BODIPY-1b**, and (j–l) **BODIPY-2b** with different magnifications (500 ×, 2000 ×, and 5000 ×).

**Figure 6:**
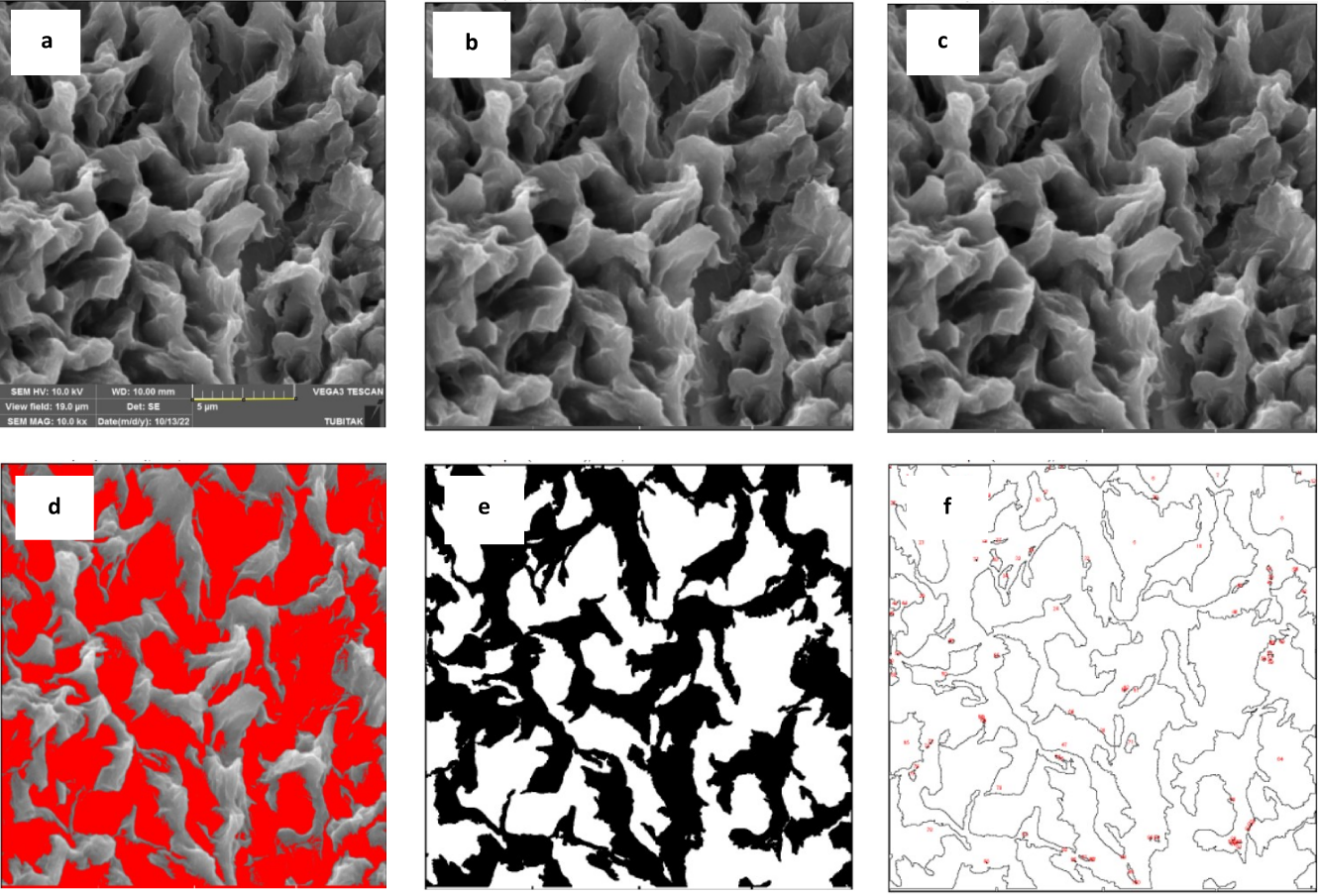
(a) Sequence of image analysis of **BODIPY-2b** by ImageJ.^46^ (b) Micrograph of SEM that is imported to ImageJ. (c) Convert to 8-bit type for better calculation. (d) Adjust threshold for better calculation. (e) Apply binary-fill holes and binary-erode steps. (f) Calculate the amount of pores on the micrograph regarding the scale.

The morphological analyses of BODIPY molecules were performed by SEM and HR-TEM. SEM images of **BODIPY-1a** (Figure 5a–c), **BODIPY-2a** (Figure 5d–f), **BODIPY-1b** (Figure 5g–i), and **BODIPY-2b** (Figure 5j–l) are displayed in Figure 5 with different magnifications (500 ×, 2000 ×, 5000 ×). SEM images of **BODIPY-1a**, **BODIPY-2a,** and **BODIPY-1b** showed plate-like structures, which correspond to crystalline structures, whereas the image of **BODIPY-2b** displayed sponge-like structures with a porosity of 51%, determined by ImageJ (Figure 6). SEM-EDS of BODIPY derivatives was also done to show the percentage of elements present (Figure S22).

**Figure 7:**
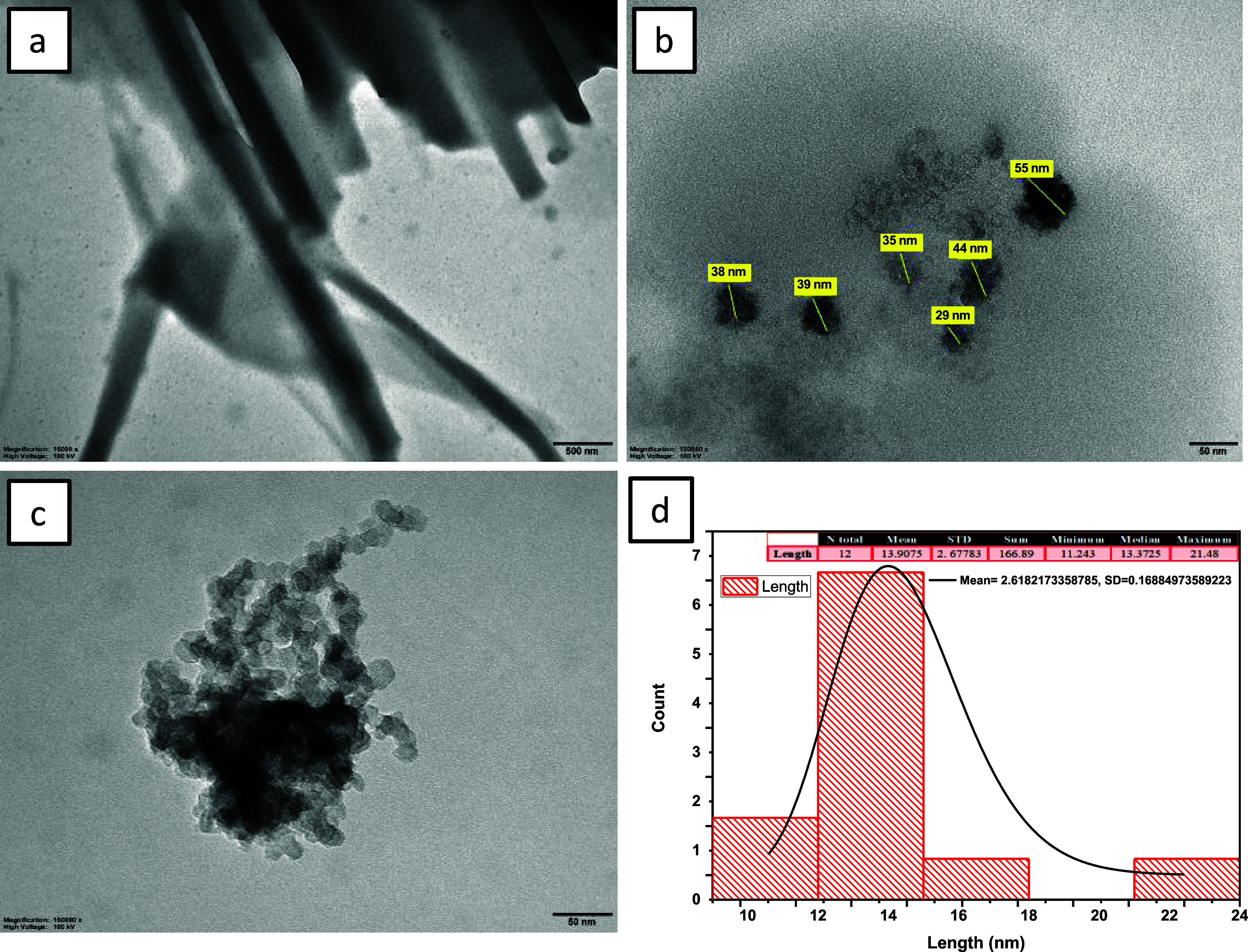
HR-TEM images of **BODIPY-1b** (a) as bulk and (b) as solid formed in NMR tube via slow evaporation of CDCl_3_ and (c) **BODIPY-2b** and (d) size distribution of **BODIPY-2b**.

HR-TEM images of **BODIPY-1b** and **BODIPY-2b** are displayed in Figure 7. An arrangement could not be observed for bulk **BODIPY-1b** in the HR-TEM analysis (Figure 7a). However, a spherical structure was observed for **BODIPY-1b** solids formed in the NMR tube via slow evaporation of CDCl_3_ (c = 2 × 10^–1^ M) (Figure 7b). An overlapped spherical structure with an average diameter of 13.37 nm was observed for **BODIPY-2b** (Figure 7c, d).

### X-Ray Diffraction Analysis

**Figure 8:**
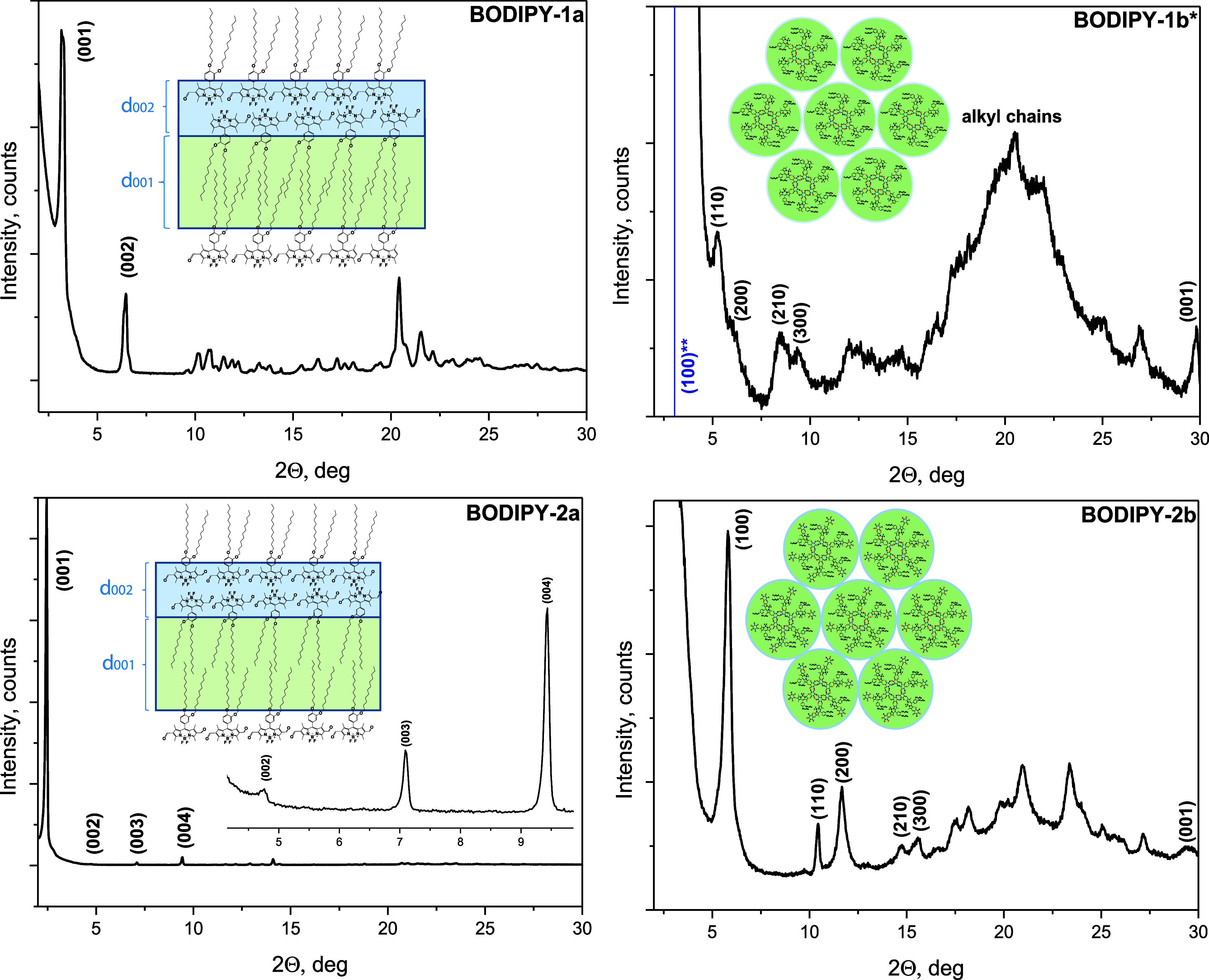
XRD patterns of BODIPY derivatives and their proposed possible arrangements. *Data belong to **BODIPY-1b** solids obtained by slow evaporation of CDCl_3_ solution (2 × 10^–1^ M) in an NMR tube. **Calculated value of d_100_ for **BODIPY-1b**.

The organization of BODIPY derivatives was investigated by X-ray diffraction (XRD) measurements on bulk powder samples of **BODIPY-1a**, **BODIPY-2a**, and **BODIPY-2b** and solids of **BODIPY-1b** obtained by slow evaporation of CDCl_3_ solution (c = 2 × 10^–1^ M) in an NMR tube (Figure 8). All XRD data are summarized in Table 1. XRD pattern of bulk **BODIPY-1b** could not be evaluated due to high background (Figure S23). The powder diffraction patterns of **BODIPY-1a** and **BODIPY-2a** contain typical reflections of lamellar crystalline structure with the ratio of 1:2 and 1:2:3:4, respectively, in the low angle region. The layer-to-layer distance (d_001_) of **BODIPY-2a** is higher than the layer-to-layer distance (d_001_) of **BODIPY-1a.**

**Table 1:** X-ray Diffraction Data of BODIPY Derivatives at Room Temperature^a^

**Compound**	**Structure**	**d**_**obs**_**(Å)**	*d*_**calc**_**(Å)**	**Ratio**	**Miller indices (hkl)**	**Lattice parameter (Å)**
**BODIPY-1a**	Lamellar	27.4599	27.4599	1	(001)	
		13.6683	13.7299	2	(002)	
**BODIPY-2a**	Lamellar	35.9562	35.9562	1	(001)	
		18.5481	17.9781	2	(002)	
		12.4576	11.9854	3	(003)	
		9.3672	8.9891	4	(004)	
BODIPY-1b*	Hexagonal	-	28.9886	1	(100)	a = 33.47
		16.7366	16.7366	√3	(110)	
		14.5110	14.4943	√4	(200)	
		10.3480	10.9567	√7	(210)	
		9.3724	9.6629	√9	(300)	
		2.99			(001)	
**BODIPY-2b**	Hexagonal	15.2107	15.2107	1	(100)	a = 17.56
		8.4578	8.7819	√3	(110)	
		7.5725	7.6054	√4	(200)	
		6.0004	5.7491	√7	(210)	
		5.6850	5.0702	√9	(300)	
		3.02			(001)	

a Data belong to **BODIPY-1b** solids obtained by slow evaporation of CDCl_3_ solution (2 × 10^–1^ M) in an NMR tube.

The powder diffraction patterns of **BODIPY-1b**, obtained by slow evaporation of CDCl_3_ solution (c = 2 × 10^–1^ M) in an NMR tube, and **BODIPY-2b** displayed reflections with the ratio of 1:√3:√4:√7:√9, suggesting a two-dimensional hexagonal lattice with disc-like molecules stacked in columns in the hexagonal arrangement. These results support the formation of cyclic rosettes via hydrogen bonding.

## Experimental Section

### Materials and Instruments

All chemicals were purchased from Sigma-Aldrich, Merck, Acros Organics, Alfa Aesar, and Fluka. The infrared spectra were acquired with a PerkinElmer Spectrum100 FT-IR spectrometer equipped with an attenuated total reflection (ATR) accessory containing a zinc selenide (ZnSe) crystal. Matrix-assisted laser desorption/ionization time-of-flight mass spectrometry (MALDI-TOF-MS) measurements were obtained with a Bruker Daltonics MicrOTOF mass spectrometer. All NMR spectra were recorded in deuterated solvents on a Varian INOVA 500 MHz spectrometer at 298 K with tetramethylsilane (TMS) as an internal reference, and chemical shift values (δ) were given in ppm. A Shimadzu UV2600 spectrophotometer was used to record UV–vis absorption spectra. Fluorescence spectra were measured using a Varian Eclipse spectrofluorometer with 1.0 cm path length cuvettes at room temperature. Dynamic light scattering (DLS) experiments were done using a Zetasizer analyzer (Model: Malvern Zetasizer Nano-ZS) instrument. The surface morphology and energy-dispersive X-ray (EDX) analysis were recorded with an FEI (PHILIPS) XL30 SFEG scanning electron microscope. Transmission electron microscopy (TEM) images were obtained using a Hitachi HT7700 with an EXALENS (120 kV) in high-resolution (HR) mode. Powder X-ray diffraction measurements were carried out on a Bruker Advanced D8 X-ray diffractometer with a Cu Kα (λ = 1.5405 Å) radiation source operating at 30 kV and 30 mA.

### Synthesis of BODIPY-1a

Anhydrous DMF (1 mL) was placed under an argon atmosphere in a two-neck round-bottom flask placed in an ice bath. POCl_3_ (1 mL) was added and then stirred for 30 min at room temperature. A pale yellow, viscous solution was obtained. Compound **2** (100 mg, 0.15 mmol) was dissolved in 10 mL of 1, 2-dichloroethane (DCE) and then slowly added to the mixture with a dropping funnel. The mixture was set to 68 °C, stirred for an additional 2 h, and then cooled to room temperature. It was poured into an ice-cold saturated NaHCO_3_ solution. It was warmed to room temperature, extracted twice with DCM, and dried over anhydrous Na_2_SO_4._ Suction filtration was applied and then evaporated. It was purified by silica gel column chromatography using ethanol/DCM (v/v = 1/100) as the eluent. An orange-colored product was obtained. Yield: 71 mg (0.10 mmol, 68%). FT-IR [(ATR) ν_max_/cm^–1^]: 2296–2856 (aromatic C–H), 2756 (aldehyde C–H), 1664 (C=O). MALDI-TOF-MS (DIT): *m*/*z* Calcd: 720.82, Found: 720.17 [M]^+^, 701.05 [M-F]^+^. ^1^H NMR (500 MHz, CDCl_3_, ppm): δ 10.0 (s, 1H, CHO), 7.0 (d, 1H, Ar–CH, ^3^J_H–H_ = 7.99 Hz), 6.8–6.9 (m, 2H, Ar–CH), 6.1 (s, 1H, CH), 4.1 (t, 2H, OCH_2_, ^3^J_H–H_ = 6.7 Hz), 3.9 (t, 2H, OCH_2_, ^3^J_H–H_ = 6.7 Hz), 2.8 (s, 3H, CH_3_), 2.6 (s, 3H, CH_3_), 1.8–1.9 (m, 4H, CH_2_), 1.8 (s, 3H, CH_3_), 1.5 (s, 3H, CH_3_) 1.2–1.3 (m, 36H, CH_2_), 0.8–0.9 (m, 6H, CH_3_). ^13^C NMR (125 MHz, CDCl_3_, ppm): δ 186.0, 161.4, 156.4, 150.1, 150.0, 147.4, 143.8, 143.0, 134.4, 130.1, 126.3, 126.1, 123.8, 120.2, 113.8, 112.9, 69.6, 69.3, 31.9, 29.7, 29.7, 29.7, 29.6, 29.6, 29.5, 29.4, 29.4, 29.3, 29.2, 26.1, 25.9, 22.7, 15.1, 14.9, 14.1, 13.0, 11.7.

### Synthesis of BODIPY-2a

In a two-neck round-bottom flask placed on an ice bath, anhydrous DMF (0.5 mL) was added under argon. POCl_3_ (0.5 mL) was added and then stirred for 30 min at room temperature. A pale yellow viscous solution was obtained. The compound **BODIPY-1a** (60 mg, 0.08 mmol) was dissolved in 5 mL of 1,2-dichloroethane (DCE) and then slowly added to the mixture with a dropping funnel. The mixture was set to 65 °C, stirred for an additional 2 h, and then cooled to room temperature. The brownish solution was poured into an ice-cold saturated NaHCO_3_ solution. It was warmed up to room temperature, then extracted with water twice, and dried over anhydrous Na_2_SO_4_. Suction filtration was applied and then evaporated. It was purified by silica gel column chromatography using ethanol/DCM (v/v = 1/100) as the eluent. An orange-colored product was obtained. Yield: 30 mg (0.04 mmol, 62%). FT-IR [(ATR) ν_max_/cm^–1^]: 2922–2854 (aliphatic C–H), 2720 (aldehyde C–H), 1673 (C=O). MALDI-TOF-MS (DHB): *m*/*z* Calcd: 748.83 g/mol, Found: 749.95 [M + H]^+^, 730.16 [M+H–F]^+^. ^1^H NMR (500 MHz, CDCl_3_, ppm): δ 10.1 (s, 2H, CHO), 7.0 (d, 1H, Ar–CH, ^3^J_H–H_ = 7.9 Hz), 6.7–6.8 (m, 2H, Ar–CH), 4.1 (t, 2H, OCH_2_, ^3^J_H–H_ = 6.7 Hz), 4.0 (t, 2H, OCH_2_, ^3^J_H–H_ = 6.7 Hz), 2.9 (s, 6H, CH_3_), 1.8–1.9 (m, 2H, CH_2_), 1.8 (s, 6H, CH_3_), 1.3–1.5 (m, 38H, CH_2_), 0.8–0.9 (m, 6H, CH_3_). ^13^C NMR (125 MHz, CDCl_3_, ppm): δ 185.7, 160.5, 150.6, 150.4, 148.4, 147.6, 132.2, 128.0, 127.9, 127.8, 125.3, 119.9, 114.0, 112.4, 69.7, 69.2, 31.9, 31.9, 29.7, 29.7, 29.6, 29.6, 29.6, 29.5, 29.4, 29.4, 29.2, 29.2, 26.1, 26.0, 22.7, 22.6, 14.1, 13.7, 12.2.

### Synthesis of BODIPY-1b

The compound **BODIPY-1a** (73 mg, 0.1 mmol) and barbituric acid (132 mg, 1 mmol) were stirred in absolute ethanol (7 mL) under an argon atmosphere. The reaction mixture was refluxed overnight. The obtained product was washed with water. The pink precipitate was gathered by filtration. Yield: 39 mg (0.05 mmol, 46%). FT-IR [(ATR) ν_max_/cm^–1^]: 3200–3054 (barbituric NH), 2917–2910 (aliphatic C–H). MALDI-TOF-MS (DIT): *m*/*z*: Calcd: 830.89 g/mol, Found: 830.75 [M]^+^, 812.58 [M+H–F]^+^. ^1^H NMR (500 MHz, CDCl_3_, ppm): δ 8.4 (s, 1H, CH), 8.3 (s, 1H, NH), 8.0 (s, 1H, NH), 7.0 (d, 1H, Ar–CH, ^3^J_H–H_ = 8.10 Hz), 6.8 (m, 2H, Ar–CH), 6.1 (s 1H, CH), 4.1 (t, 2H, OCH_2_, ^3^J_H–H_ = 6.70 Hz), 4.0 (t, 2H, OCH_2_, ^3^J_H–H_ = 6.70 Hz), 2.6 (s, 3H, CH_3_), 2.6 (s, 3H, CH_3_), 1.8–1.9 (m, 4H, CH_2_), 1.6 (s, 3H), 1.5 (s, 3H, CH_3_), 1.2–1.3 (m, 36H, CH_2_), 0.8–0.9 (m, 6H, CH_3_). ^1^H NMR (500 MHz, CDCl_3_/D_2_O, ppm): δ 8.4 (s, 1H, CH), 7.0 (d, 1H, Ar–CH, ^3^J_H–H_ = 8.10 Hz), 6.8 (m, 2H, Ar–CH), 6.2 (s 1H, CH), 4.1 (t, 2H, OCH_2_, ^3^J_H–H_ = 6.70 Hz), 4.0 (t, 2H, OCH_2_, ^3^J_H–H_ = 6.70 Hz), 2.6 (s, 3H, CH_3_), 2.6 (s, 3H, CH_3_), 1.8–1.9 (m, 4H, CH_2_), 1.6 (s, 3H, CH_3_), 1.5 (s, 3H, CH_3_), 1.2–1.3 (m, 36H, CH_2_), 0.8–0.9 (m, 6H, CH_3_). ^13^C NMR (125 MHz, CDCl_3_, ppm): δ 162.7, 161.3, 159.8, 156.5, 151.0, 150.1, 150.0, 148.8, 147.3, 143.1, 134.5, 131.4, 129.5, 126.1, 123.9, 120.3, 114.0, 113.8, 112.9, 69.5, 69.2, 31.9, 29.6, 29.6, 29.4, 29.3, 29.2, 29.0, 26.1, 26.1, 22.7, 18.4, 15.1, 15.0, 14.8, 14.2, 14.1, 14.0.

### Synthesis of BODIPY-2b

The compound **BODIPY-2a** (30 mg, 0.04 mmol) and barbituric acid (56 mg, 0.4 mmol) were stirred in absolute ethanol (10 mL) under an argon atmosphere. The reaction mixture was refluxed overnight. The obtained product was washed with water. The purple precipitate was gathered by filtration. Yield: 31 mg (0.03 mmol, 79%). FT-IR [(ATR) ν_max_/cm^–1^]: 3201–3027 (barbituric NH), 2923–1153 (aliphatic C–H). MALDI-TOF-MS (DIT): *m*/*z* Calcd 968.97 g/mol, Found: 969.19 [M]^+^, 992.19 [M + Na]^+^, 950.26 [M-F]^+^. ^1^H NMR (500 MHz, THF-*d*_*8*_, ppm): δ 10.4 (s, 2H, NH), 10.3 (s, 2H, NH), 8.2 (s, 2H), 7.1 (d, 1H, Ar–CH, ^3^J_H–H_ = 8.3 Hz), 7.0 (s, 1H, Ar–CH), 6.8–6.9 (m 1H, Ar–CH), 4.0 (t, 2H, OCH_2_, ^3^J_H–H_ = 6.5 Hz), 3.9 (t, 2H, Ar–CH, ^3^J_H–H_ = 6.5 Hz), 2.5 (s, 6H, CH_3_), 1.7–1.8 (m, 4H, CH_2_), 1.5 (s, 6H, CH_3_), 1.2–1.3 (m, 36H, CH_2_), 0.8–0.9 (m, 6H, CH_3_). ^1^H NMR (500 MHz, THF-*d*_*8*_/D_2_O, ppm): δ 8.2 (s, 2H), 7.1 (d, 1H, Ar–CH, ^3^J_H–H_ = 8.3 Hz), 7.0 (s, 1H, Ar–CH), 6.8–6.9 (m 1H, Ar–CH), 4.0 (t, 2H, OCH_2_, ^3^J_H–H_ = 6.5 Hz), 3.9 (t, 2H, Ar–CH, ^3^J_H–H_ = 6.5 Hz), 2.5 (s, 6H, CH_3_), 1.7–1.8 (m, 4H, CH_2_), 1.5 (s, 6H, CH_3_), 1.2–1.3 (m, 36H, CH_2_), 0.8–0.9 (m, 6H, CH_3_). ^13^C NMR (125 MHz, THF-*d*_8_, ppm): δ 162.3, 160.6, 158.1, 150.6, 150.4, 149.7, 149.6, 145.4, 144.3, 144.0, 132.2, 127.6, 126.2, 120.1, 119.8, 113.8, 113.2, 68.9, 68.7, 31.9, 29.7, 29.7, 29.6, 29.4, 26.1, 26.1, 24.8, 22.6, 13.9, 13.5, 13.3.

## Conclusion

Hydrogen bonding is an important driving force in the self-assembly of molecules, and barbituric acid is a very suitable tool to build hydrogen-bonded architectures prepared by an easy synthetic procedure. Novel barbituric acid-substituted BODIPY derivatives were synthesized via the Knoevenagel condensation between formyl BODIPY and barbituric acid in ethanol solution without a catalyst under reflux conditions in moderate yield. The aggregation behavior of the dyes was evaluated either in a polar solvent, ethanol, or a nonpolar solvent mixture, THF-MCH. Blueshifted absorption indicating H-type aggregation and a gradual increase of emission were observed in ethanol over time. Redshifted absorption indicating parallel J-type aggregation and an anti-Stokes shift were observed in the THF-MCH mixture. Concentration-dependent ^1^H NMR confirmed a hydrogen bonded cyclic arrangement in CDCl_3_. Furthermore, the self-assembly of molecules was also investigated in the solid state via SEM, HR-TEM, and PXRD. Formyl derivatives of BODIPYs show a lamellar arrangement, while XRD of barbituric acid-substituted BODIPYs shows a two-dimensional hexagonal lattice with disc-like molecules stacked in columns in the hexagonal arrangement. These results provide compelling evidence for the formation of cyclic rosettes via hydrogen bonding interactions. To the best of our knowledge, this work represents the first demonstration of cyclic rosettes involving BODIPY, thereby paving the way for novel developments in BODIPY chemistry and its applications.

## Supporting Information Available

The spectra corresponding to the characterization of BODIPY compounds, additional spectrophotometric graphs related to UV–vis, SEM-EDS of BODIPY compounds, and powder XRD of bulk **BODIPY-1b** (PDF)
