# Supplementary material for: Tuning the Photophysical Properties of BODIPY Dyes and Studying Their Self-Assembly via Hydrogen Bonding
Source: ACS Omega. 2024 Dec 27;10(1):1716–26. doi: 10.1021/acsomega.4c09745 (PMC11740828; doi:10.1021/acsomega.4c09745)
Supplement: Supplementary file 1 — ao4c09745_si_001.pdf [file ao4c09745_si_001.pdf]

SUPPORTING INFORMATION

Tuning the photophysical properties of BODIPY dyes and studying their self-assembly via hydrogen bonding

Büşra Akyol, Eylül Merve Çokluk, Mehmet Menaf Ayhan, Sinem Tuncel Kostakoğlu* and Ayşe Gül Gürek*

Gebze Technical University, Department of Chemistry, Gebze 41400, Kocaeli, Turkey

Supporting Information

Contents

1. FT-IR, NMR, and Mass Spectra of Compounds

2. Absorption Spectroscopy

3. SEM-EDS Analysis of Compounds

4. XRD Analysis

1. FT-IR, NMR, and Mass Spectra of Compounds

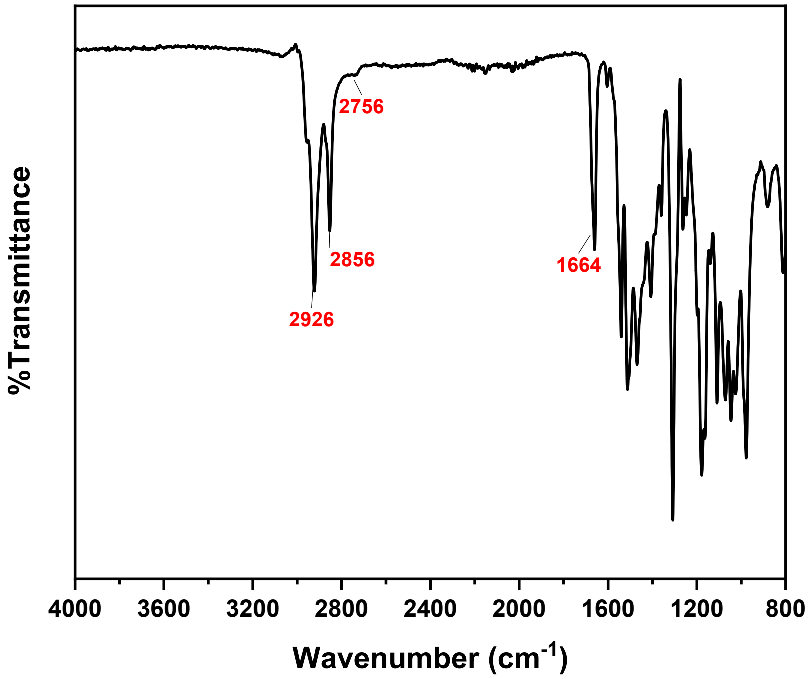

Fig. S1 FT-IR spectrum of compound **BODIPY-1a**.

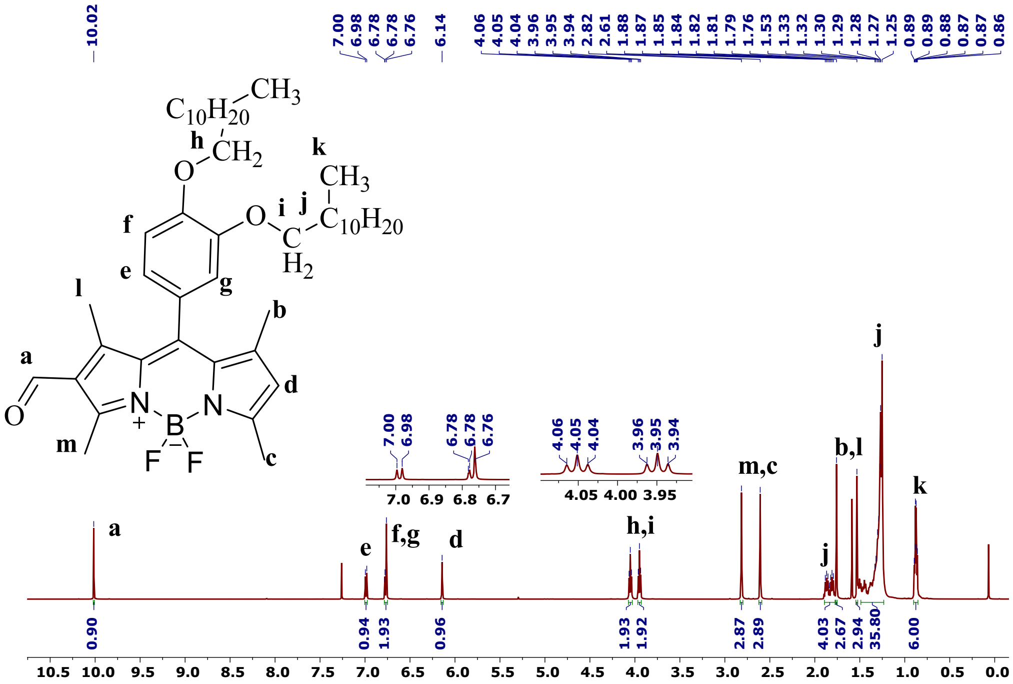

Fig. S2 ^{1}H-NMR (500 MHz) spectrum of compound **BODIPY-1a** in $CDCl_3$.

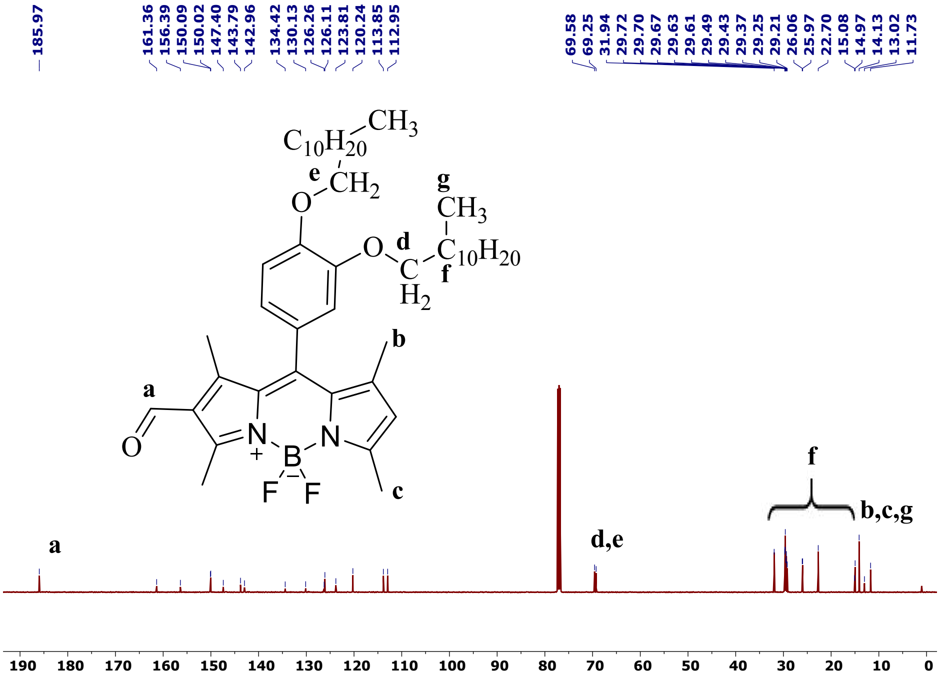

Fig. S3 ^{13}C-NMR (125 MHz) spectrum of compound **BODIPY-1a** in $CDCl_3$.

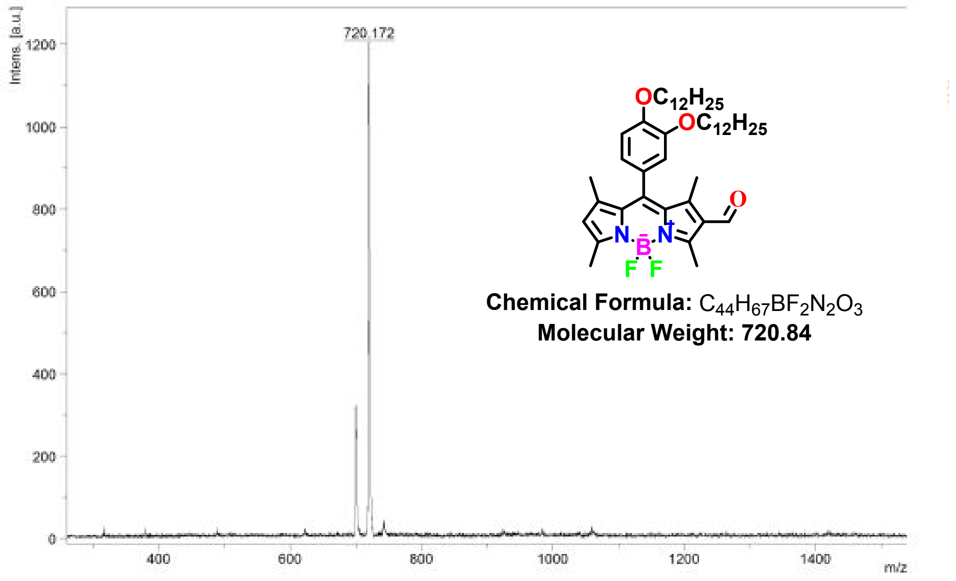

Fig. S4 MALDI-TOF-MS spectrum of compound **BODIPY-1a**.

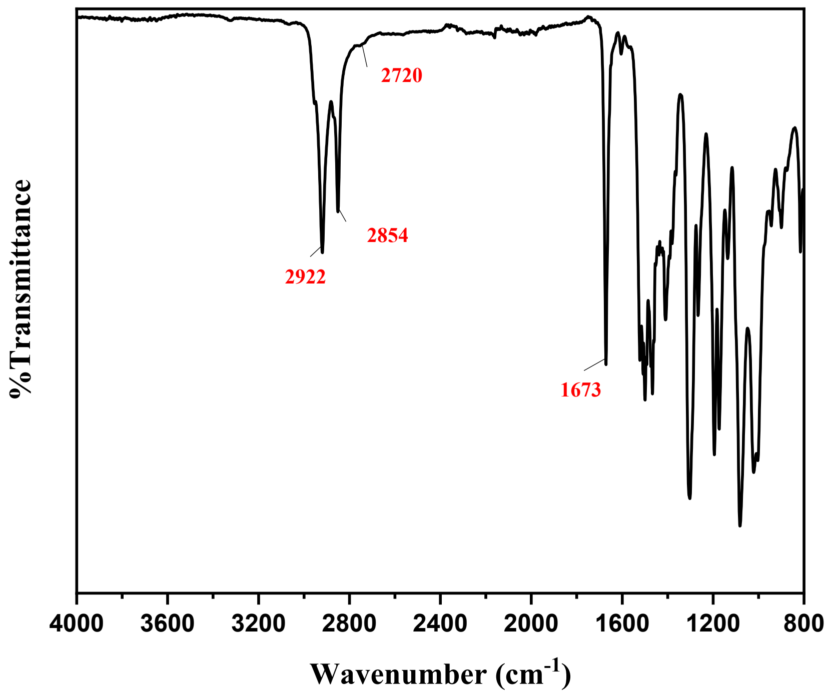

Fig. S5 FT-IR spectrum of compound **BODIPY-2a**.

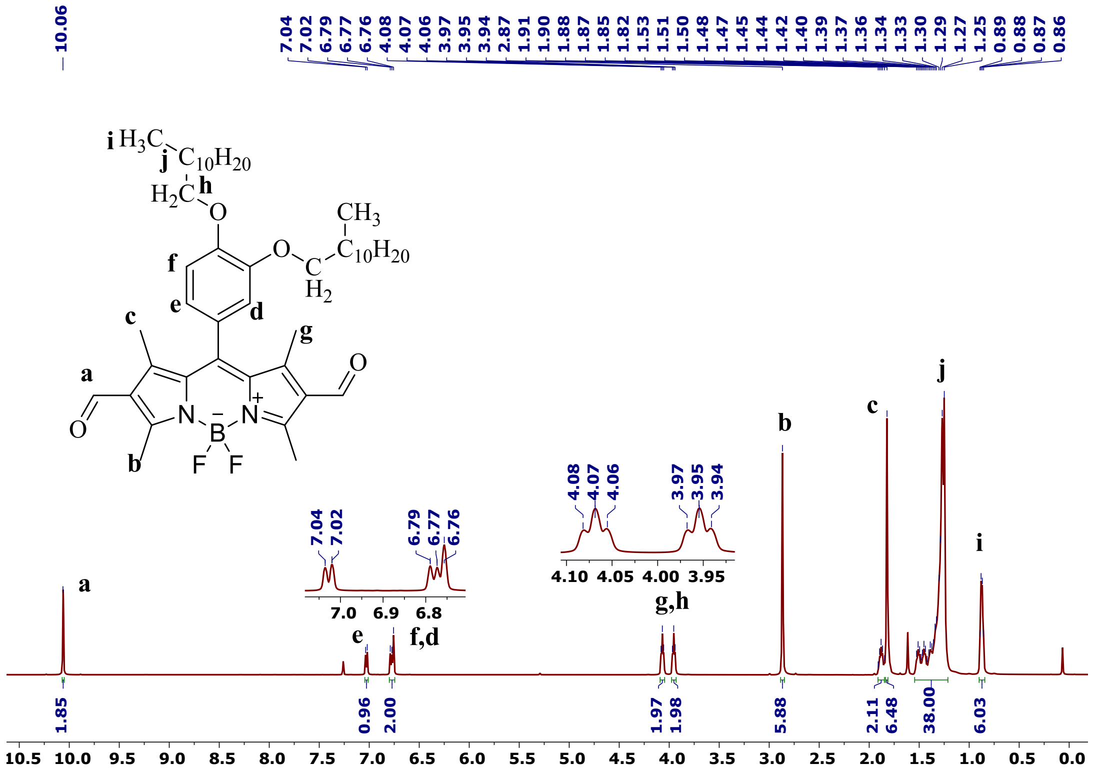

Fig. S6 ^{1}H-NMR (500 MHz) spectrum of compound **BODIPY-2a** in $CDCl_3$.

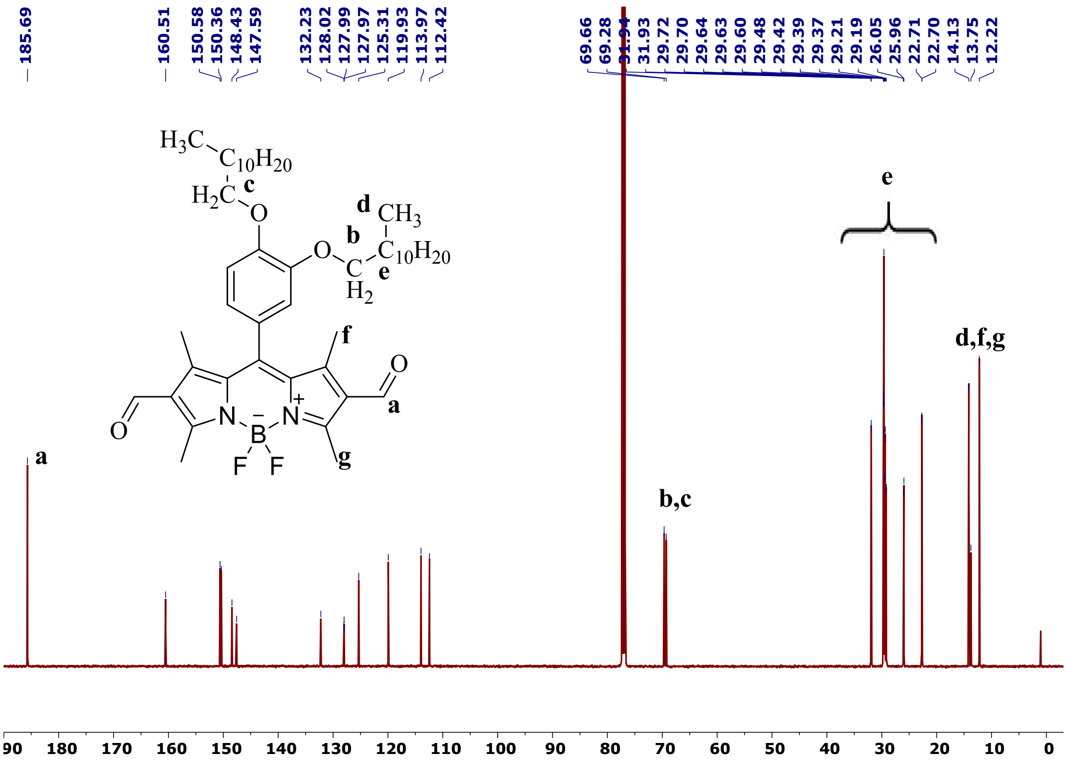

Fig. S7 ^{13}C-NMR (125 MHz) spectrum of compound **BODIPY-2a** in $CDCl_3$.

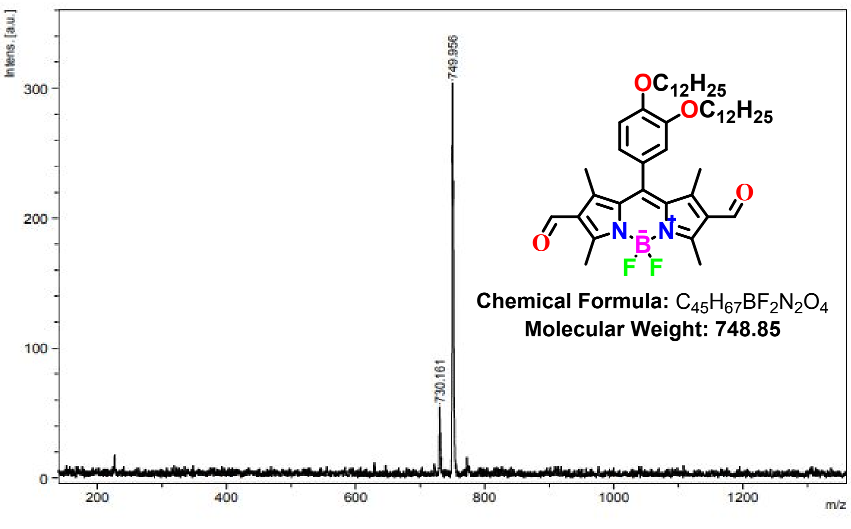

Fig. S8 MALDI-TOF-MS spectrum of compound **BODIPY-2a**.

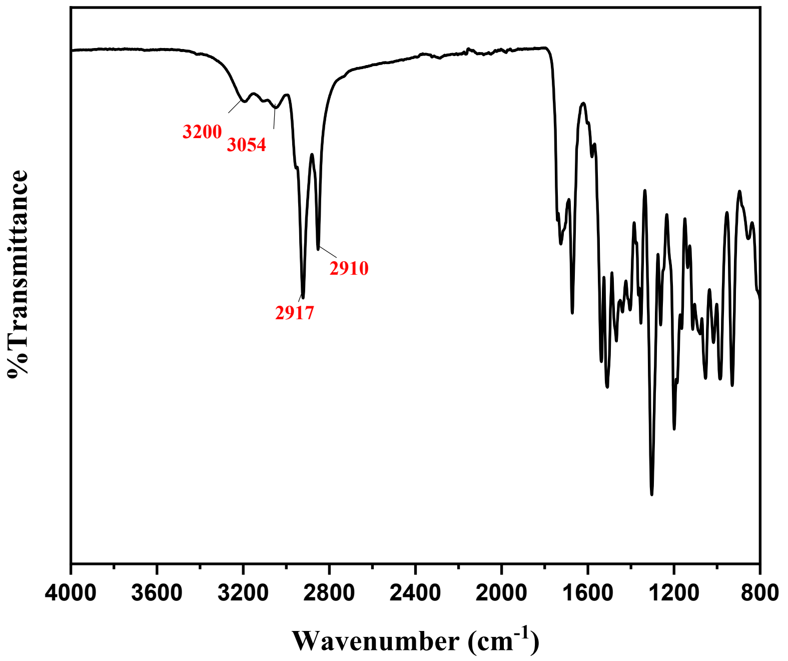

Fig. S9 FT-IR spectrum of compound **BODIPY-1b**.

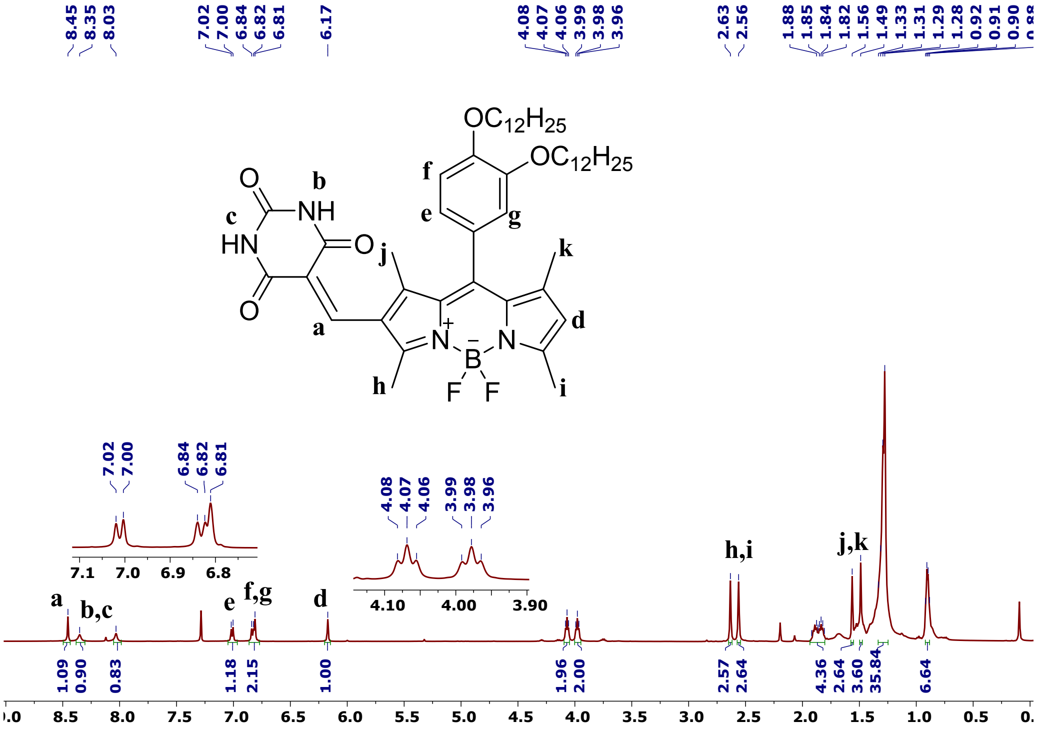

Fig. S10 ^{1}H-NMR (500 MHz) spectrum of compound **BODIPY-1b** in $CDCl_3$.

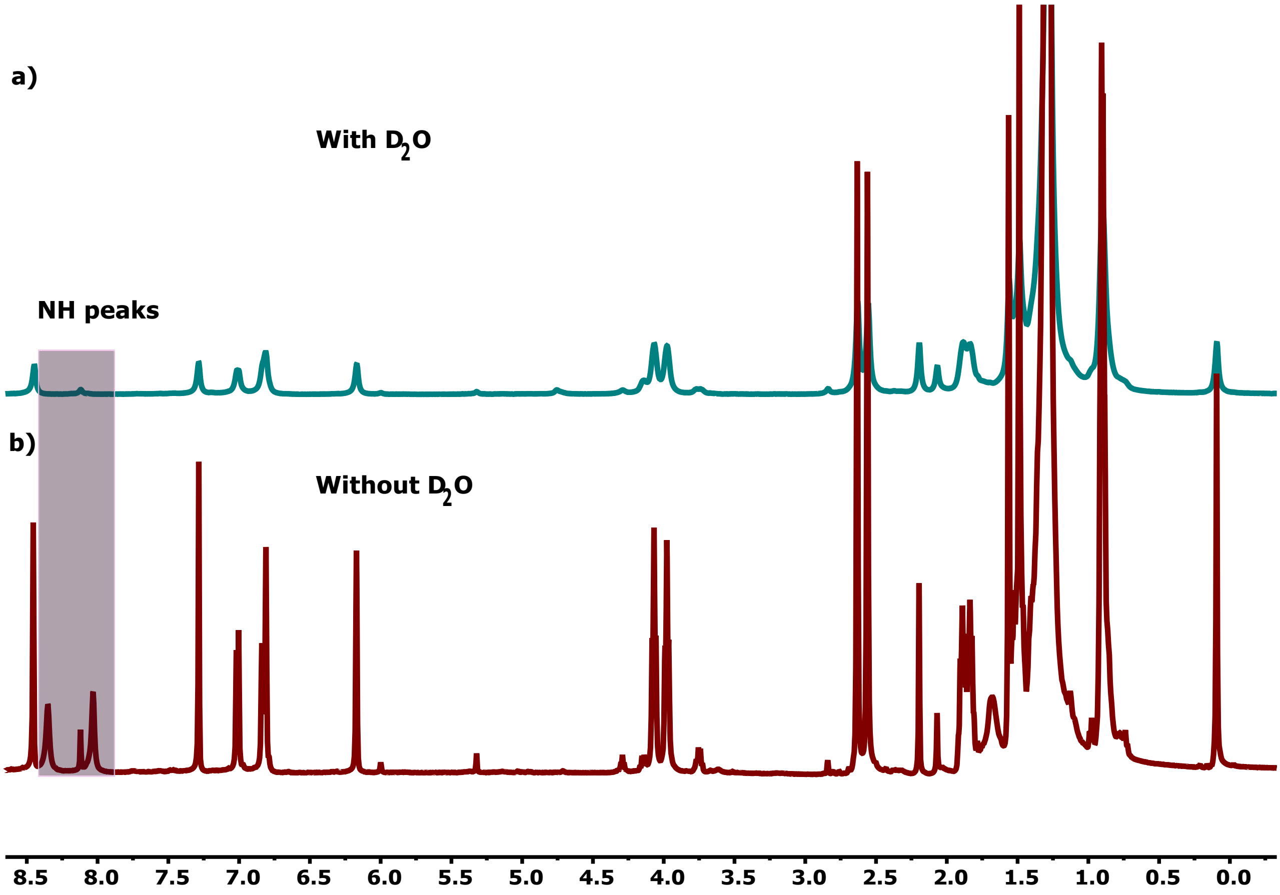

Fig. S11 ^{1}H-NMR (500 MHz) spectrum of compound **BODIPY-1b** (a) in $CDCl_3$ and D_2O, (b) in $CDCl_3$.

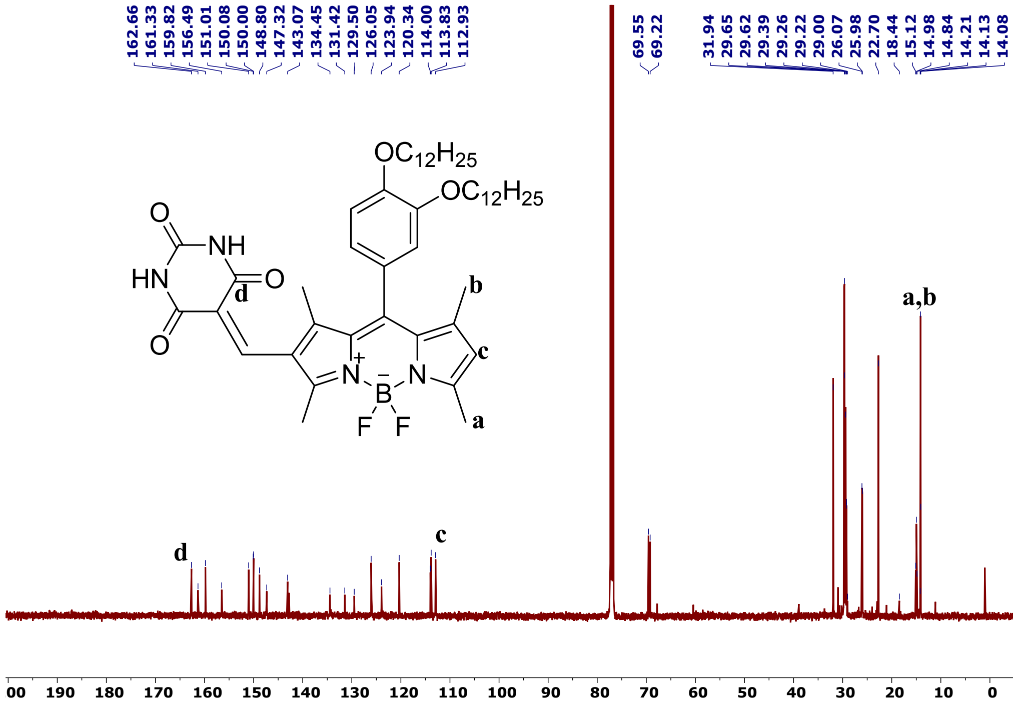

Fig. S12 ^{13}C-NMR (125 MHz) spectrum of compound **BODIPY-1b** in $CDCl_3$.

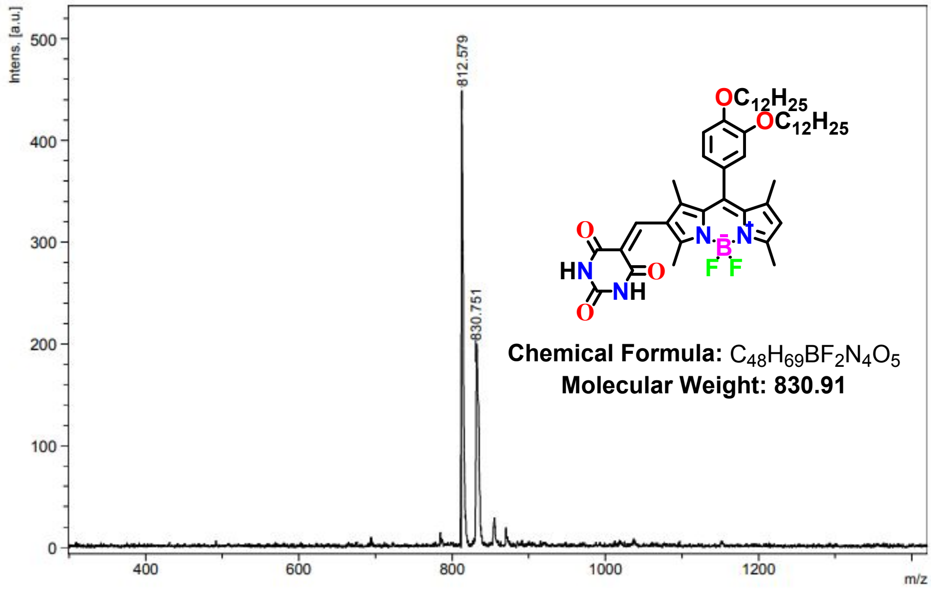

Fig. S13 MALDI-TOF-MS spectrum of compound **BODIPY-1b**.

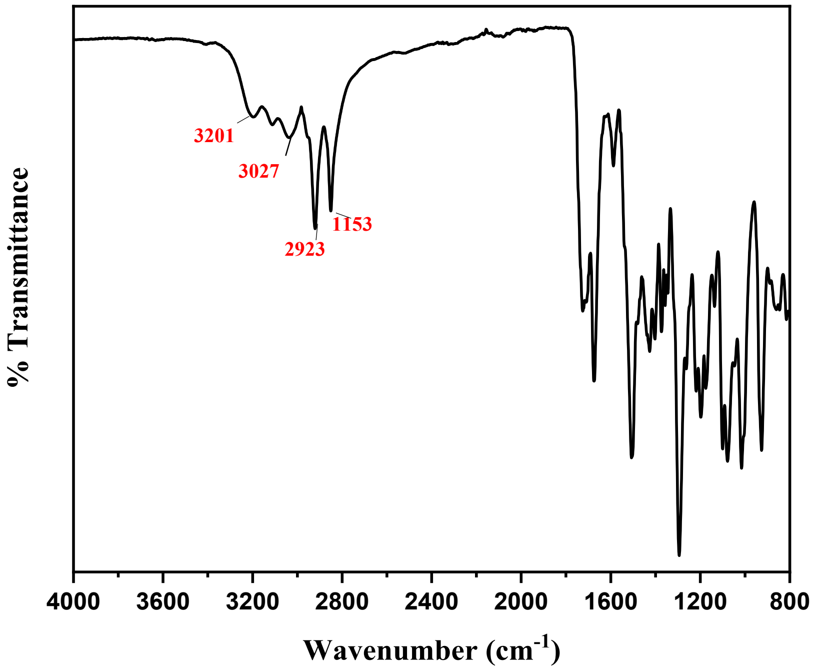

Fig. S14 FT-IR spectrum of compound **BODIPY-2b**.

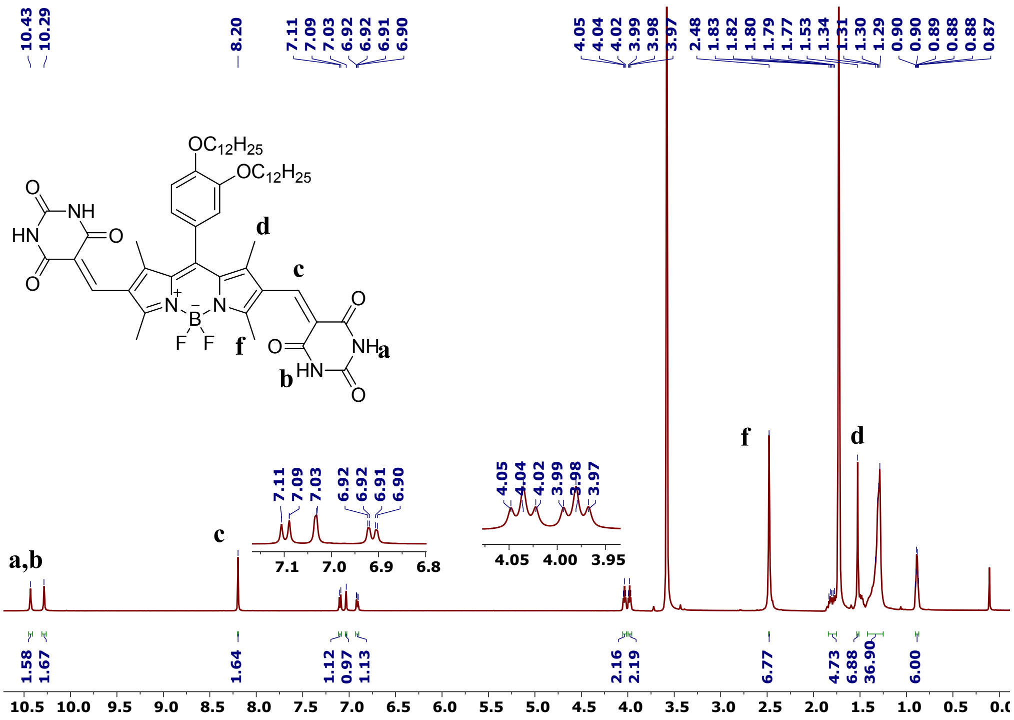

Fig. S15 ¹H- NMR (500 MHz) spectrum of compound **BODIPY-2b** in THF-d_8.

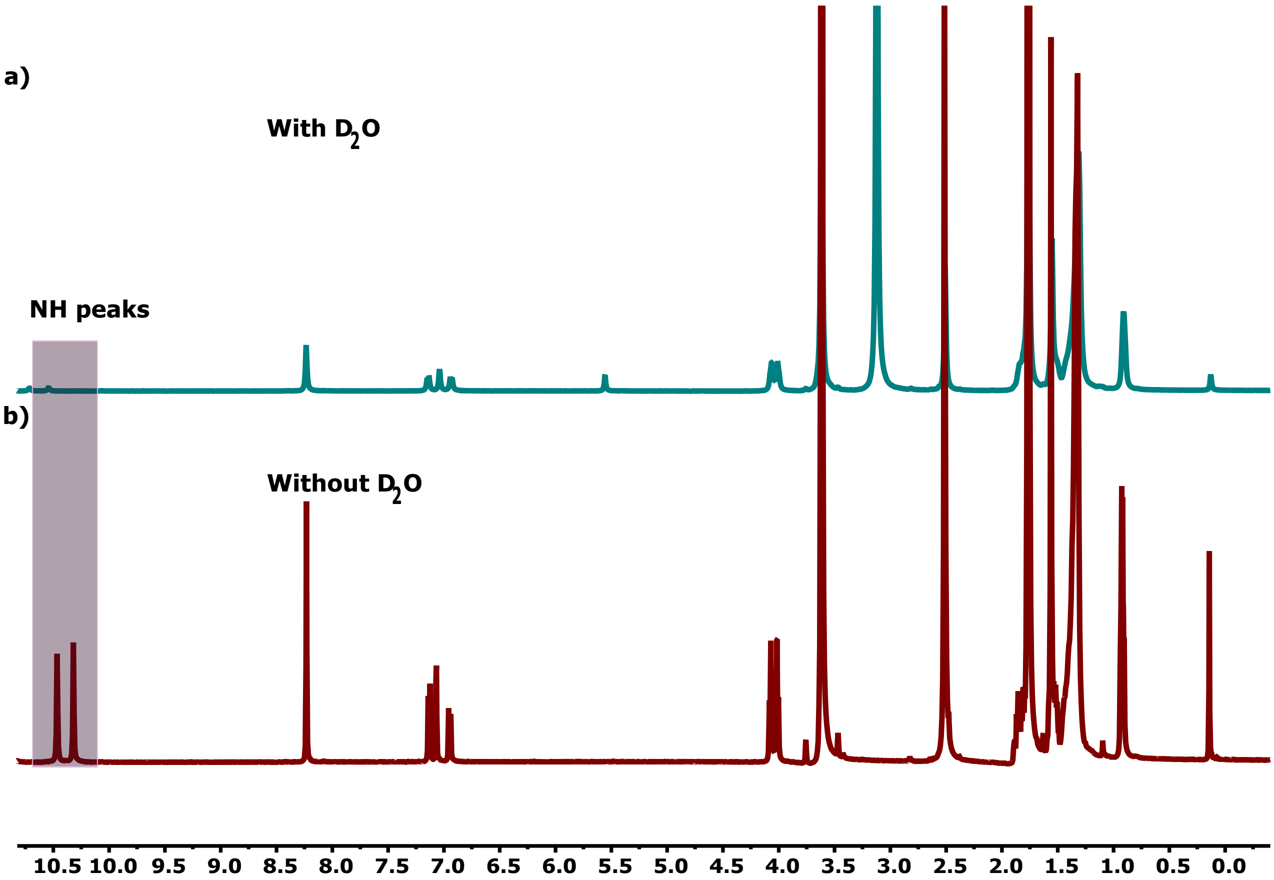

Fig. S16 ¹H-NMR (500 MHz) spectrum of compound **BODIPY-2b** (a) in THF-d_8 and D_2O, (b) in THF-d_8.

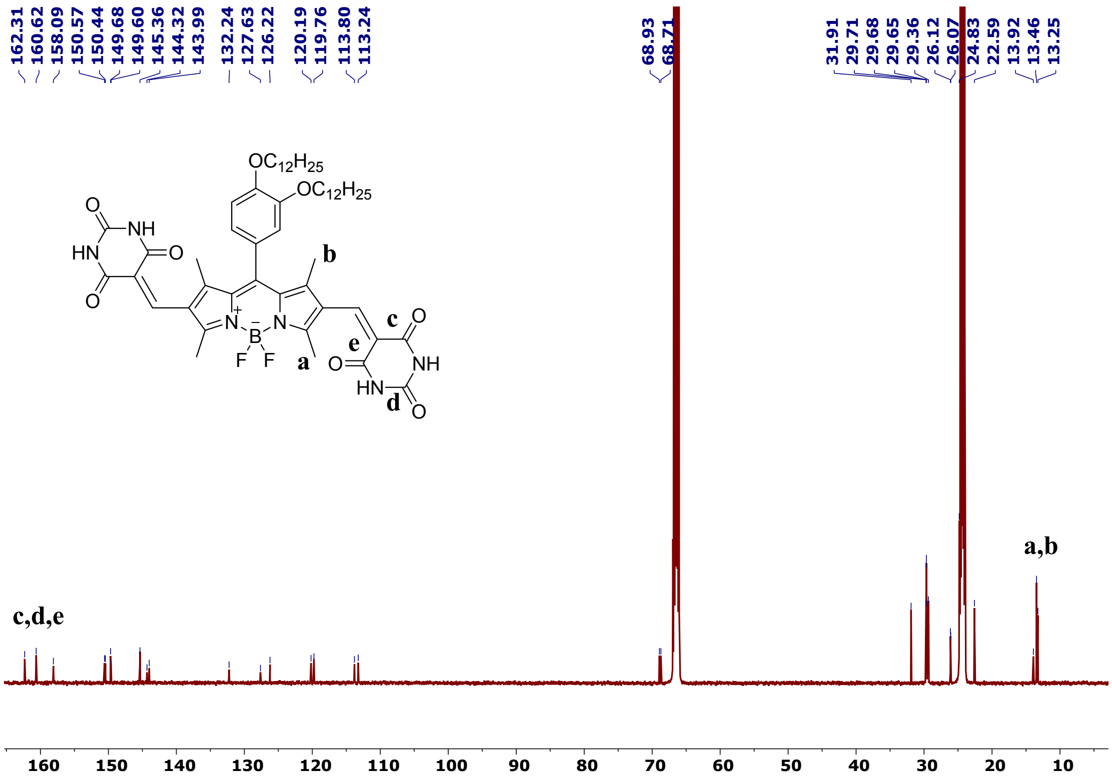

Fig. S17 ^{13}C-NMR (125 MHz) spectrum of compound **BODIPY-2b** in THF-d_8.

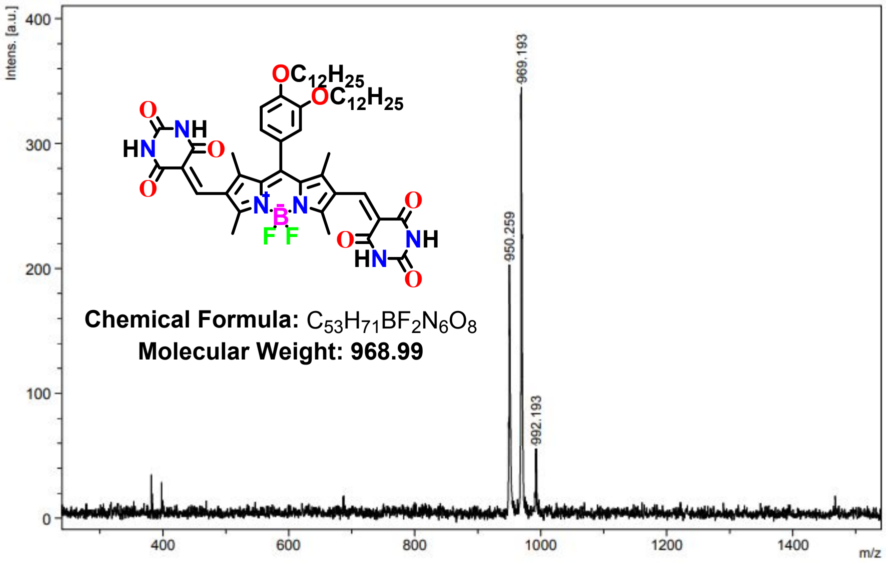

Fig. S18 MALDI-TOF-MS spectrum of compound **BODIPY-2b**.

2. Absorption Spectroscopy

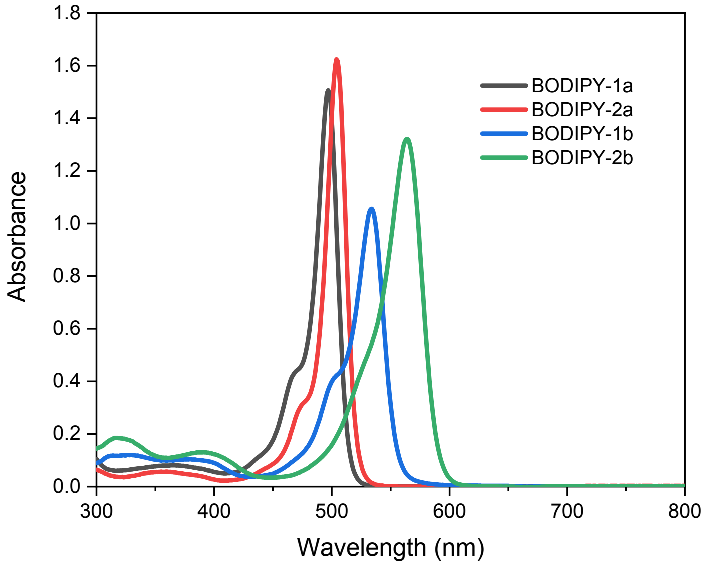

Fig. S19 UV-Vis spectra of compounds **BODIPY-1a**, **BODIPY-2a**, **BODIPY-1b,** and **BODIPY-2b** in THF (10 μM).

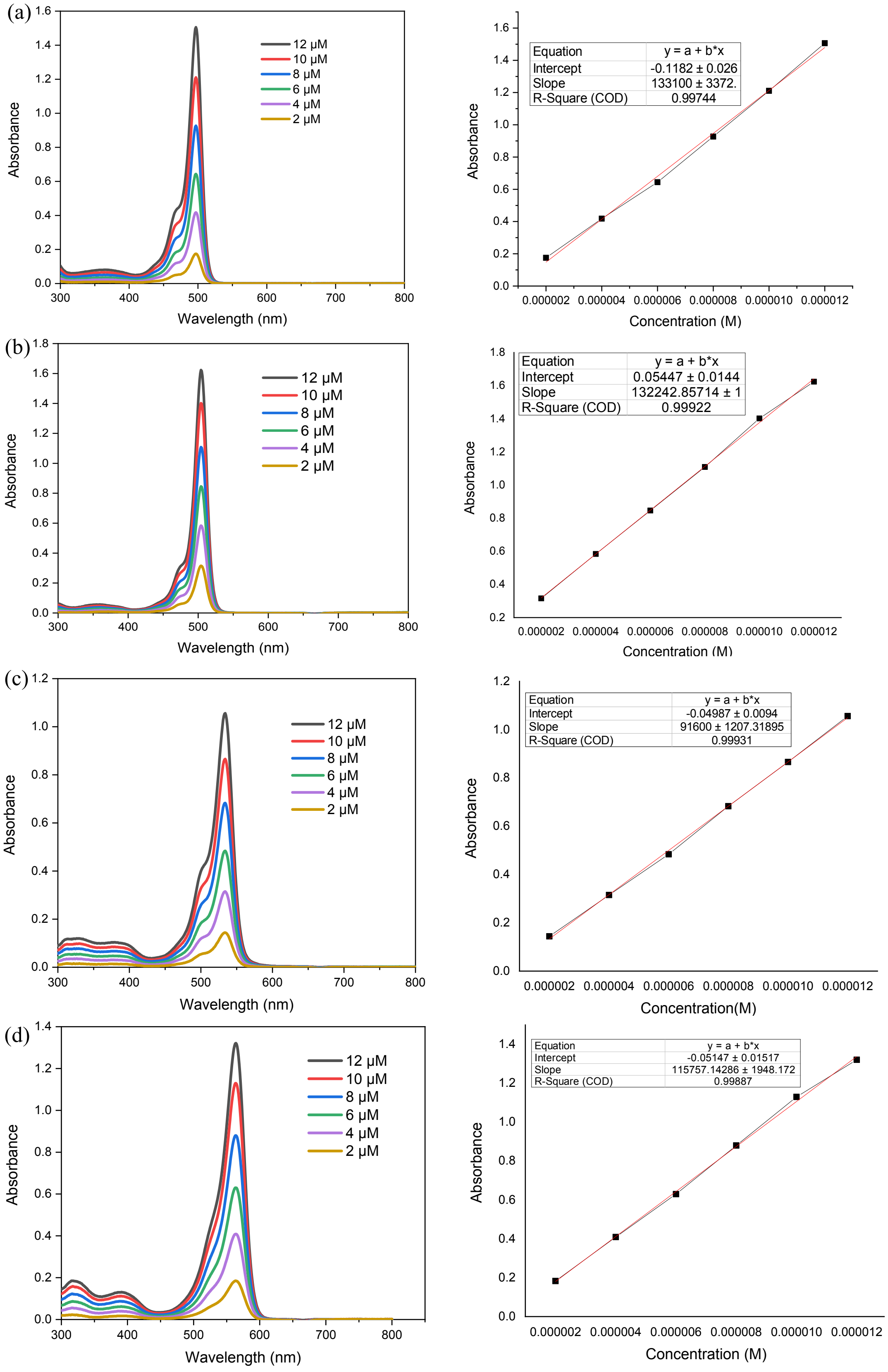

Fig. S20 UV-Vis spectra of compounds a) **BODIPY-1a**, b) **BODIPY-2a**, c) **BODIPY-1b,** and d) **BODIPY-2b** in THF at different concentrations.

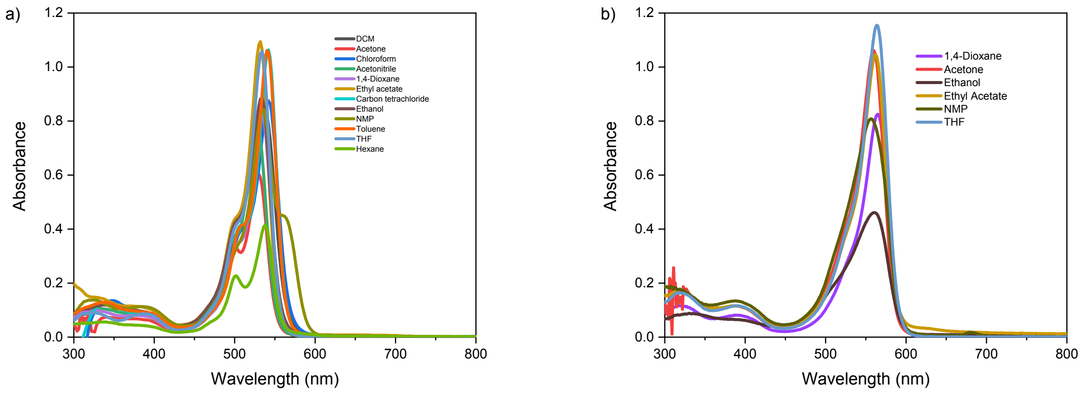

Fig. S21 Solvent dependent UV-Vis studies of (a) **BODIPY-1b** and (b)**BODIPY-2b** in different solvents ($c=1x10^{-5}$ M).

3. SEM-EDS Analysis of Compounds

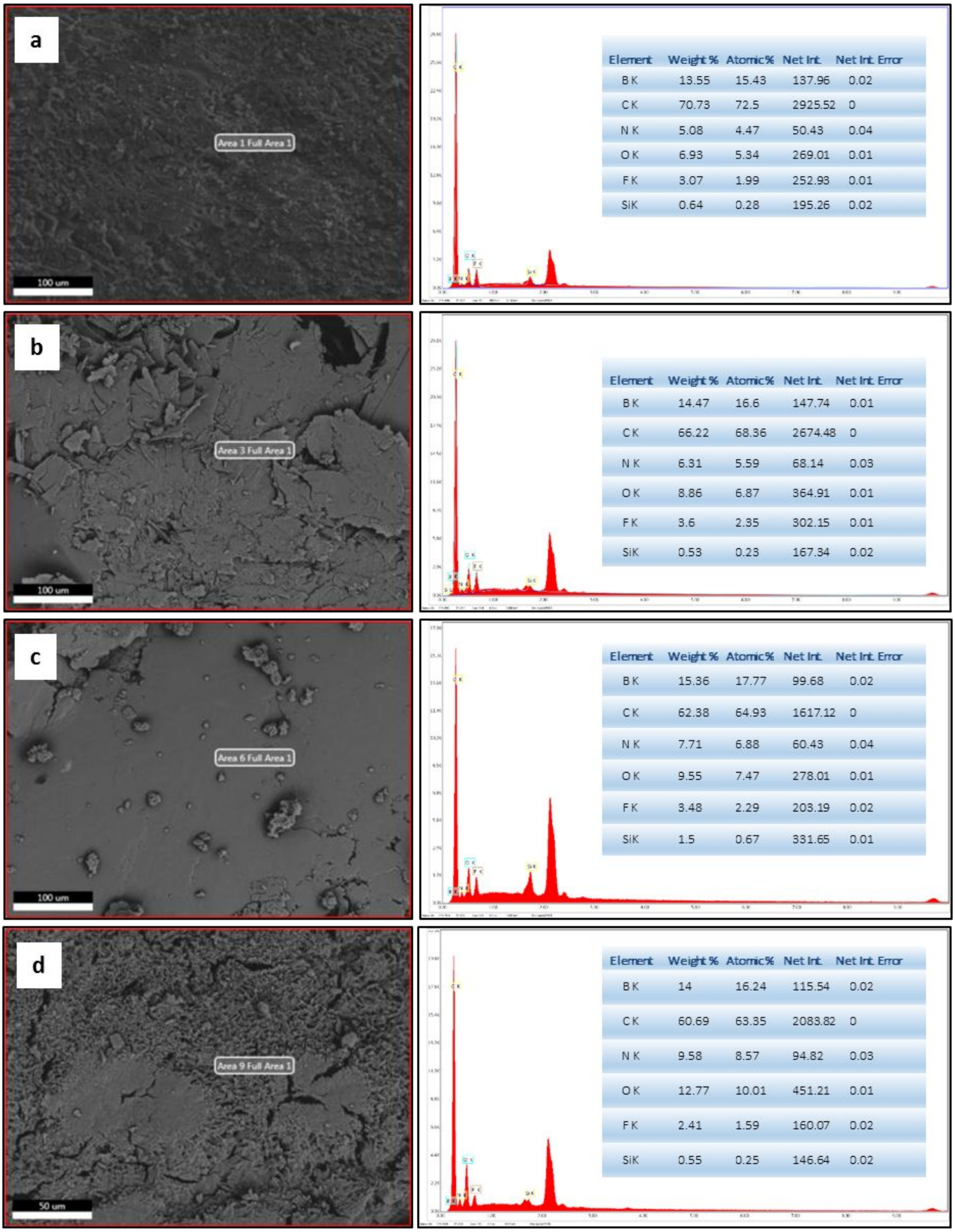

Fig. S22. SEM-EDS analysis of **BODIPY-1a** (a), **BODIPY-2a** (b), **BODIPY-1b** (c) and **BODIPY-2b** (d).

4. XRD Analysis

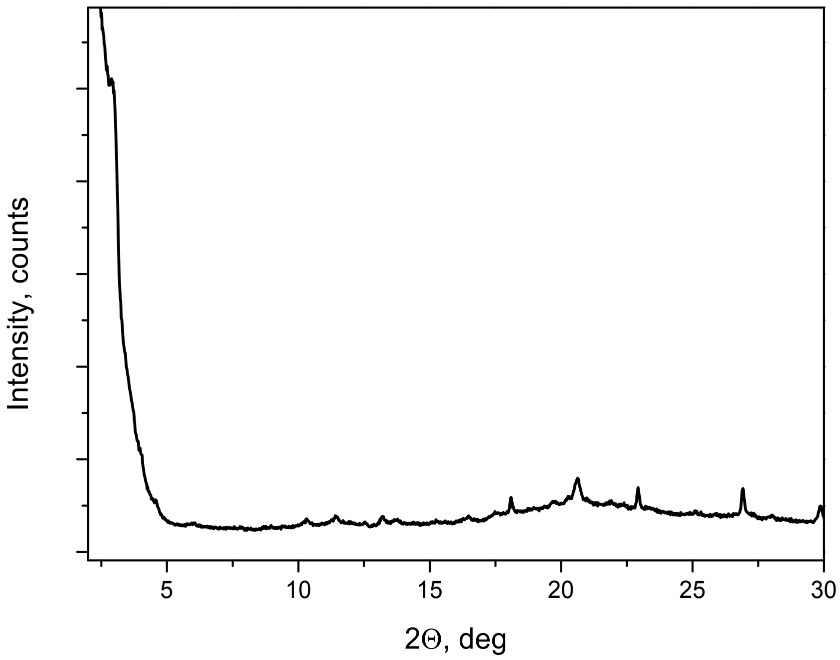

Fig. S23. XRD pattern of untreated **BODIPY-1b**.
